# Multidimensional behavioral profiles associated with resilience and susceptibility after inescapable stress

**DOI:** 10.1038/s41598-024-59984-7

**Published:** 2024-04-27

**Authors:** Benedito Alves de Oliveira-Júnior, Danilo Benette Marques, Matheus Teixeira Rossignoli, Tamiris Prizon, João Pereira Leite, Rafael Naime Ruggiero

**Affiliations:** Department of Neuroscience and Behavioral Sciences, Ribeirão Preto Medical School, University of São Paulo, Ribeirão Preto, São Paulo Brazil

**Keywords:** Animal models, Depression, Anxiety, Stress coping, Multivariate analysis, Machine learning, Anxiety, Depression, Stress and resilience

## Abstract

Clinical depression is characterized by multiple concurrent symptoms, manifesting as a complex heterogeneous condition. Although some well-established classical behavioral assessments are widespread in rodent models, it remains uncertain whether rats also display stress-induced depression-related phenotypes in a multidimensional manner, i.e., simultaneous alterations in multiple behavioral tests. Here, we investigated multivariate patterns and profiles of depression-related behavioral traits in male Wistar rats subjected to inescapable footshocks (IS) or no-shocks (NS), followed by a comprehensive battery of behavioral tests and ethological characterization. We observed generalized stronger intra-test but weaker inter-test correlations. However, feature clustering of behavioral measures successfully delineated variables linked to resilience and susceptibility to stress. Accordingly, a noteworthy covariation pattern emerged, characterized by increased open field locomotion, reduced time in the elevated plus maze open arms, lower sucrose preference, and increased shuttle box escape failures that consistently differentiated IS from NS. Surprisingly there is little contribution from forced swim. In addition, individual clustering revealed a diversity of behavioral profiles, naturally separating NS and IS, including subpopulations entirely characterized by resilience or susceptibility. In conclusion, our study elucidates intricate relationships among classical depression-related behavioral measures, highlighting multidimensional individual variability. Our work emphasizes the importance of a multivariate framework for behavioral assessment in animal models to understand stress-related neuropsychiatric disorders.

## Introduction

Major depressive disorder is the leading cause of disability worldwide, affecting more than 320 million people^1^, and imposing a substantial economic burden^2^. Clinical depression is diagnosed and characterized by the simultaneous presentation of multiple symptoms, spanning affective, motivational, somatic, sensorimotor, and cognitive domains, which manifests as complex and symptomatically heterogeneous conditions^3^. A considerable portion of patients do not respond or respond only after several different treatment attempts^4^. Given the necessity for more precise therapeutic strategies, the delineation of distinct clinical biotypes holds promise for enhancing treatment accuracy and efficacy^5,6^. Hence, understanding the etiological mechanisms underlying this symptomatic diversity is imperative for developing more effective therapeutic interventions. In this context, modeling these complex clinical semiologies in animal models is essential^7^.

The majority of studies investigating aspects of depression in animal models focus on stress-induced effects^8–10^. Among these models, the learned helplessness model, characterized by impaired escape performance following inescapable shocks, has been established as a valid paradigm affecting a constellation of psychobiological domains with translational relevance to depression^11,12^. Classical behavioral tests, such as the forced swim (FST), sucrose preference (SPT), elevated plus maze (EPM), open field (OF), and social interaction (SIT) are extensively employed in animal models as assessments of depression-like and anxiety-like behaviors due to their established predictive validity, verified by their sensitivity to stress and antidepressants. However, for translational research to account for the heterogeneity of clinical disorders, modern initiatives such as the Research Domain Criteria (RDoC^13,14^) advocate that experimental models should not attempt to reproduce diagnoses such as “depression” or “anxiety”, but rather, that distinct behavioral measures are related to specific *dimensions* that represent specific underlying neurobiological systems (e.g., arousal, positive valence, negative valence, sensorimotor, cognitive, and social processes), and should therefore be investigated independently at first and then examined how multiple dimensions can be linked, forming *multidimensional* associations. At the same time, these eminent depression-related behavioral variables quantified in these tests can also be embodied in the common broad construct of *resilience*, the adaptive coping or recovery from stress, versus *susceptibility*, the manifestation of stress-induced—usually maladaptive—alterations^15^. However, because these tests are commonly studied separately, it remains unclear whether rodents manifest depression-related susceptibility phenotypes in a multidimensional manner (e.g., low social interaction, anhedonia, and helplessness simultaneously) similar to the concurrent manifestation of multiple symptoms in humans.

In the literature, there is a notable unclearness in findings regarding the relationships between behavioral variables associated with depression in rodents. For instance, Strekalova et al.^16^ reported an association between sucrose preference and immobility in the FST, but Stepanichev et al.^17^ observed no correlation. Additionally, Kompagne et al.^18^ showed that chronic mild stress increased measures of social anxiety but decreased innate anxiety. In another important example, Brown et al.^19^ reported no association between shuttle box (SB) shock-escape performance and FST escape behavior. In this sense, the punctual use of few behavioral tests to distinguish depressive-related phenotypes seems to generate inconsistent results and may not be able to capture more multifaceted profiles. To address this issue, a multivariate behavioral assessment using a broad set of behavioral tests can be a valuable approach. Multivariate analysis allows for the extraction of intrinsic patterns from data that are not captured by group averages or are not obvious through conventional analysis. For decades, various multivariate analysis techniques have been used to provide a richer ethological characterization of animal behavior^20–24^. Examples of how some of these techniques have been applied to animal behavior include: (1) to reduce dimensionality and identify components or factors that represent the individual or combined contribution of variables to the phenotype, such as factor analysis and principal component analysis^21,25–27^; (2) to group variables according to similarity, such as cluster analysis^20,24, 28^, (3) to characterize the structure of behavior based on sequences of events over time, such as transition matrix analysis, adjusted residual analysis, and T-pattern analysis^22–24, 29, 30^. Such approaches have provided valuable insights into individual variability in preclinical animal models, allowing the identification of phenotypic subgroups in models of stress-related neuropsychiatric disorders and enriching their translational aspects^26–28, 31–33^. Despite the potential of these techniques, there are still few studies using this approach to assess multiple behavioral categories in test batteries^26,27, 31, 34–36^, especially in a preclinical context^27,31^. Moreover, while some studies investigated specific relationships and latent factors across behavioral dimensions, a description of the behavioral profiles that emerge in the aftermath of footshock stress is still lacking.

This study aimed to explore the associations between a wide range of behavioral variables in rats exposed to acute inescapable footshocks (IS) or no shocks (NS) and investigate patterns of covariation across these measures and how they characterize multidimensional depressive-related phenotypes. We found that feature clustering and joint variation patterns segregated behavioral variables related to susceptibility or resilience to stress. Furthermore, a covariation pattern weighting collectively on increased locomotion in the OF, reduced time in the open arms of the EPM, reduced sucrose preference, and increased escape failures in the SB, consistently differentiated IS from NS and predicted helpless rats, whereas, notably, FST behaviors had no significant relevance. Finally, we discovered distinctive phenotypic clusters that naturally discriminated IS and NS rats, including subpopulations entirely characterized by susceptibility or resilience to stress, indicating a multidimensional individual variability in stress-coping styles.

## Results

### A single-day session of acute inescapable shocks promotes long-term helplessness and discriminates between helpless (H) and not helpless (NH) individuals

**Figure 1:**
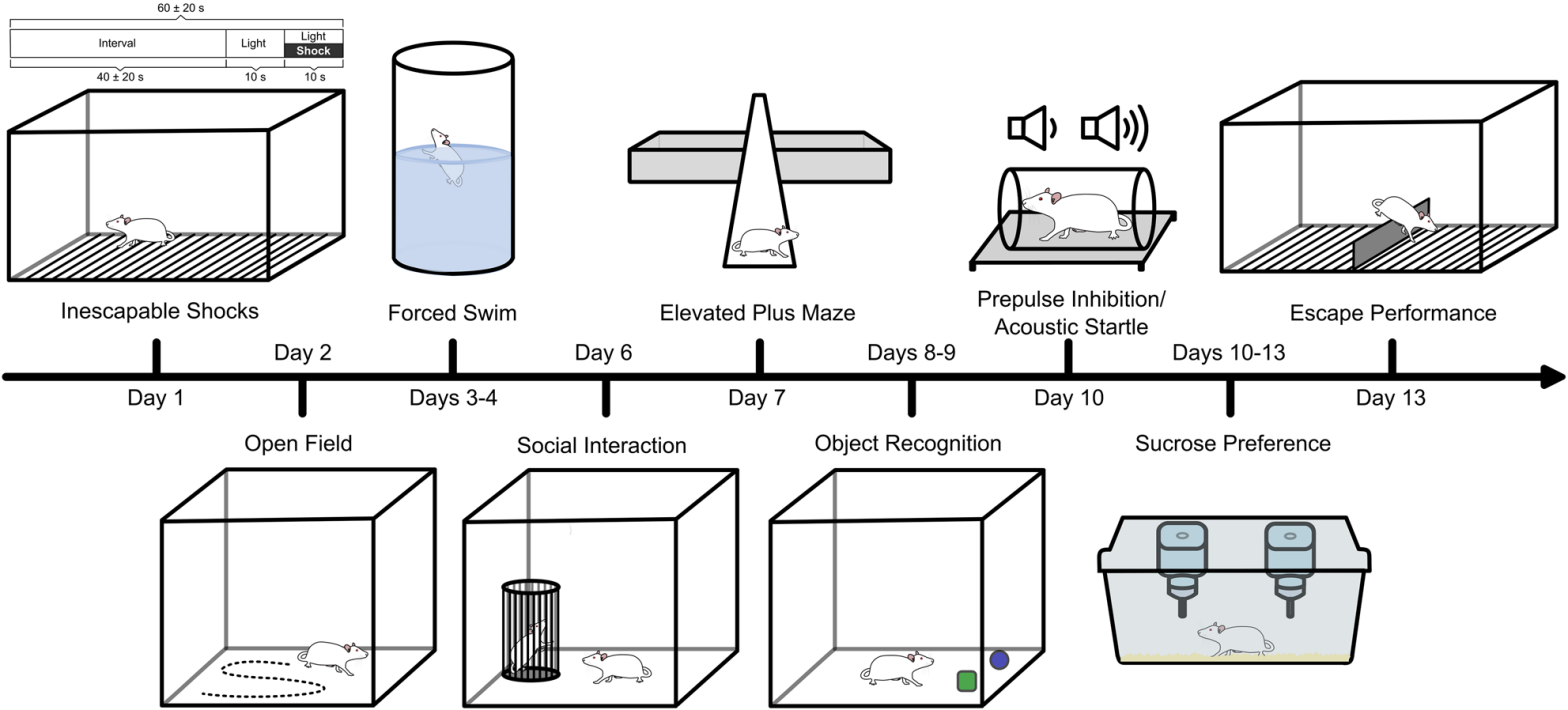
Experimental design. Schematic illustration of the inescapable footshock procedure and behavioral test battery. Illustration made with Inkscape software package^37^.

In order to investigate the relationships among diverse behavioral dimensions, we subjected rats to a comprehensive battery of behavioral tests recognized for their relevance to depression and anxiety research. These measures were evaluated following a single session of either inescapable shocks (IS) or no-shocks (NS) (Fig. 1).

**Figure 2:**
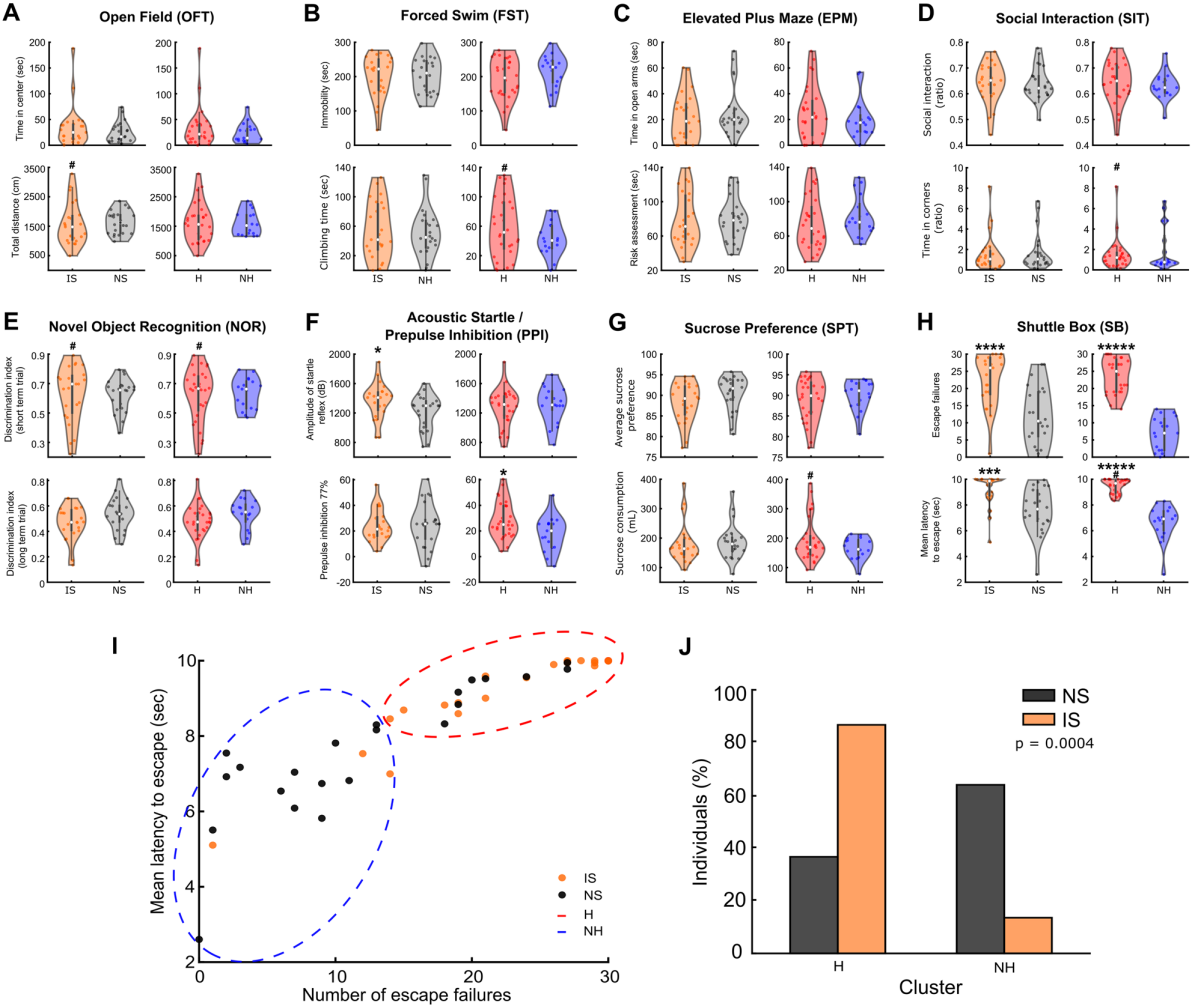
A single-day session of acute inescapable shocks promotes long-term helplessness and discriminates between helpless (H) and not helpless (NH) individuals. (**A**–**H**) Representative behavioral measures from each test. Comparison between inescapable shocks (IS, n = 23) versus no shock (NS, n = 22) groups, shown in orange and gray violin plots, respectively, and between H (n = 28) versus NH (n = 17) clusters, shown in red and blue violin plots, respectively. Note that footshocks do not promote differences between group means in most behavioral tests. However, they can increase variance in the shocked group and lead to a high proportion of helpless animals, as indicated by a high number of escape failures and high escape latency. Nevertheless, even when comparing helpless and non-helpless animals, there were no differences between group means in most behavioral tests. (**I**) Unsupervised k-means clustering of H and NH rats by number of escape failures and mean latency to escape. (**J**) Proportion of individuals from each experimental group forming the H and NH clusters. Student’s t-test: *p < 0.05; ***p < 0.001; ****p < 0.0005; *****p < 0.0001. Levene test for equality of variances: #*p* < 0.05. Data in violin plots are presented as median ± 1.5 × interquatile range.

Initially, we evaluated the univariate results by comparing the IS and NS groups (see Fig. 2). The IS group exhibited lower escape performance in the SB (escape failures: U = 693.5, *p* < 0.0001; latency to escape: U = 743, *p* = 0.0013; Fig. 2A). Furthermore, a significant difference between the groups was observed in the acoustic startle test (U = 627, *p* = 0.0342). Interestingly, no differences between groups were detected in other behavioral measures widely associated with depressive-like phenotypes (*p* > 0.05), such as immobility time in the FST (t_(43)_ =  − 0.0993, *p* = 0.9213), social interaction (t_(43)_ = 0.0618, *p* = 0.9509), or sucrose preference (t_(43)_ =  − 1.5454, *p* = 0.1295).

Noteworthy, both NS and IS groups exhibited considerable individual variability in the behaviors measured across all tests. To evaluate behavioral changes associated with helplessness, we used escape performance in the SB to classify individuals as either ‘H’ (helpless) or ‘NH’ (non-helpless). To achieve this, we employed an unsupervised clustering analysis using the k-means algorithm. The optimal number of clusters was determined to be two clusters (Fig. 2B). We found a significant difference in the number of H and NH individuals between IS and NS, in which the H cluster encompassed 51.1% of the population (n = 23/45, ${\text{X}}_{(1)}^{2}$ = 12.2443, *p* = 0.0004, chi-squared test, Fig. 2C), with the majority of these individuals (86.9%, n = 20/23) coming from the IS group. Notably, the NH cluster accounted for 48.9% of the population (n = 22/45) and primarily consisted of NS individuals (63.6%, n = 14/22).

### Feature clustering distinguishes behavioral variables associated with susceptibility and resilience to stress

**Figure 3:**
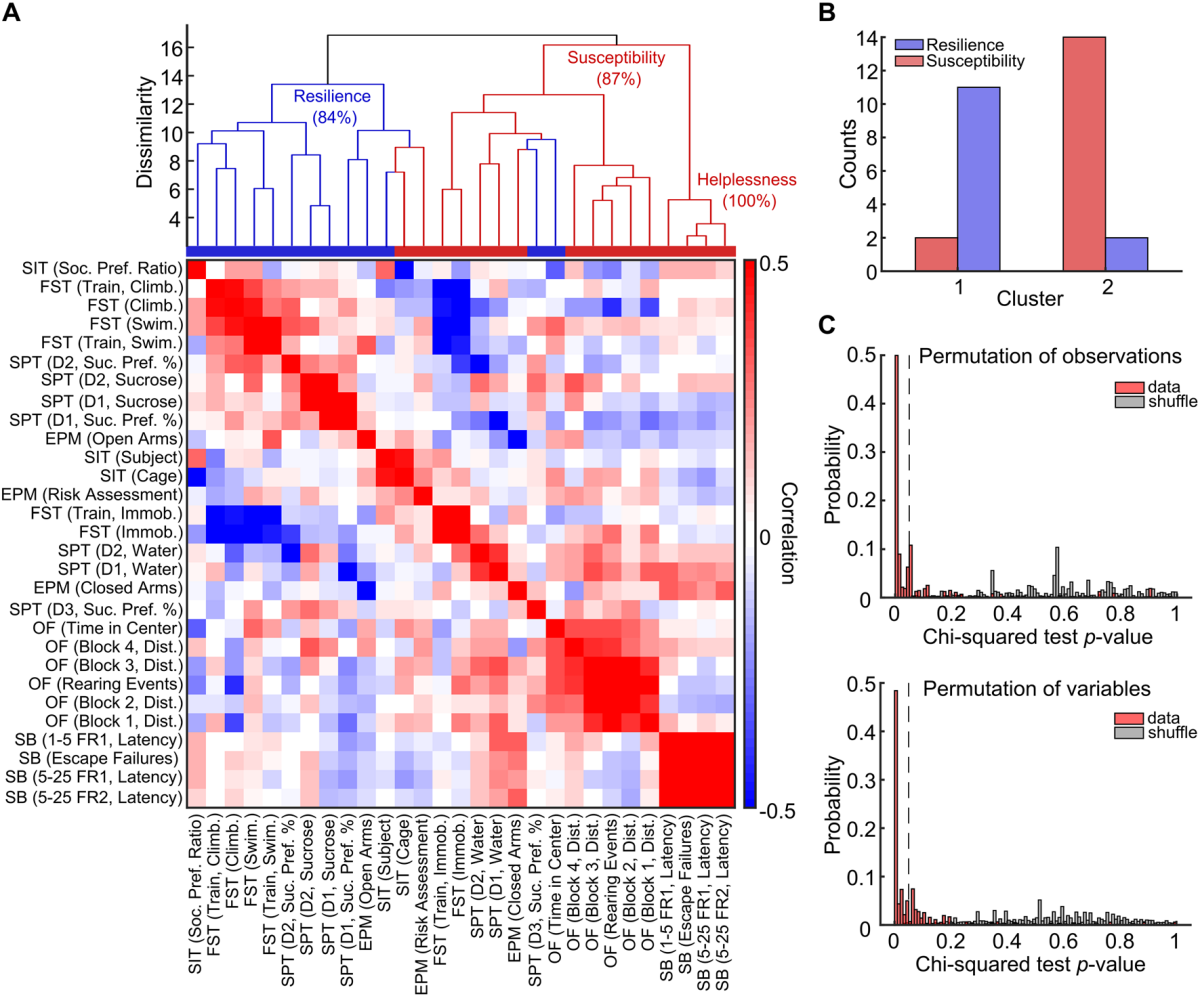
Feature clustering distinguishes behavioral variables associated with susceptibility and resilience to stress. (**A**) Hierarchical clustering of variables forms two large clusters (top) that group behavioral attributes associated with either resilience (shown in blue in the horizontal bar below the dendrogram) or susceptibility (shown in red in the same bar). The variables are clustered based on their correlation, which can be observed in more detail in the matrix (bottom). (**B**) The two large clusters are significantly consisted of susceptibility- (red) or resilience-related (blue) variables (${\text{X}}_{(1)}^{2}$ = 15.0818, *p* < 0.0001, Chi-squared test). (**C**) Distributions of resilience versus susceptibility Chi-squared test *p* values across 10^4^ iterations of randomly selecting 70% of data. This permutation approach shows that the separation between resilience versus susceptibility variables significantly occurs in most iterations even with fewer variables (top) or observations (rats; bottom) but not for shuffled data (gray bars).

To evaluate how the most relevant variables from all behavioral tests are related, we measured the correlation between them and performed hierarchical clustering. The dendrogram generated by hierarchical clustering indicates the emergence of two major clusters (Cophenetic coefficient: 0.6182, Fig. 3A). Notably, variables linked with stress resilience (see Supplementary Table 1 for the classification of behavioral variables), such as climbing in the FST and time spent in the open arms of the EPM, are grouped distinctively from those associated with stress susceptibility, such as immobility in the FST and time spent in the closed arms of the EPM. This result reveals that despite weak inter-test correlations, feature clustering could significantly assemble most susceptibility-related variables, separating them from resilience-related variables (${\text{X}}_{(1)}^{2}$ = 15.0818, *p* = 0.0001, Fig. 3B). Notably, through a data permutation approach, where fewer observations or variables were randomly selected, it was confirmed that the separation between susceptibility- and resilience-related variables occurs significantly in most of the iterations with partitioned data (permutation of observations: 62.96% significant; permutation of variables: 62.25% significant, Fig. 3C, top). In contrast, such a distinction was largely absent when analyzing shuffled data (permutation of observations: 0.15% significant; permutation of variables: 0.46% significant, Fig. 3C, bottom). These findings strongly indicate that the grouping of variables is not random but rather corresponds to two clearly defined behavioral profiles.

Meanwhile, we observed that behavioral variables are more correlated within tests than between tests, indicating that all behavioral parameters analyzed from a single test correspond to a well-defined behavioral dimension (Fig. S1). Hierarchical clustering based on sign-independent distance was able to significantly separate the behavioral tests (Cophenetic coefficient.: 0.2748; ${\text{X}}_{(25)}^{2}$ = 101.1548, *p* < 0.0001, Fig. S1A). Similarly, as shown by factor analysis, seven factors suffice to capture latent covariation corresponding to the six behavioral tests, with each factor showing high loadings only across variables of the same test (Fig. S1B,C). These results indicate that behavioral variables of a single test have a precise delimitation and represent a single behavioral domain unit, despite indicating a more comprehensive multivariate covariation related to overall resilience versus susceptibility.

### Multidimensional patterns of covariation across behavioral measures discriminate resilience and susceptibility

**Figure 4:**
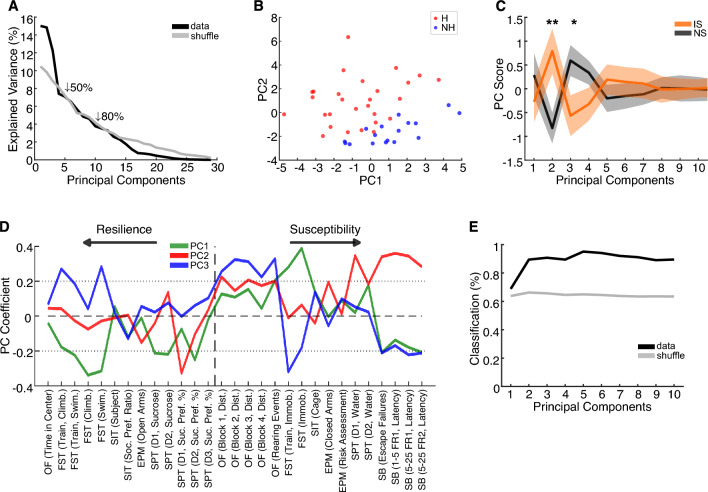
PCA reveals multidimensional patterns of covariation across behavioral measures that discriminate resilience and susceptibility. (**A**) Markedly greater explained variance by the first principal components (PCs), particularly PC1-3, than expected from shuffled data. (**B**) PCA map, which uses PC1 and PC2 scores, discriminates between H and NH individuals. (**C**) Some of the first PCs significantly distinguish NS and IS individuals. (**D**) Among the first PC coefficients, PC2 exhibits high weights in helplessness- and susceptibility-related variables (right side of the plot) and low weights in resilience-related variables (left side of the plot). Also note that PC1 and PC3 exhibit lower contributions of SB escape performance and have opposing weights on the FST, indicating independent variability of both behavioral domains. Variables are ordered by resilience and susceptibility, and then, by experimental design, following the test battery sequential order from left to right. (**E**) A linear discriminant model fitted on cumulative PC scores shows great classification performance of NH versus H compared to shuffled data. *p < 0.05; **p < 0.01. Lines and shaded boundaries represent the mean ± SEM.

Considering that variables exhibit distinct patterns of organization based on their covariation, our subsequent objective was to explore these patterns utilizing principal component analysis (PCA). This analytical technique enables the identification of dominant patterns of covariation that explain the most variance in the dataset. Furthermore, we sought to elucidate how these multivariate patterns correlate with overall resilience and susceptibility. PCA is particularly advantageous for exploring potential underlying effects in datasets with extensive information. Using all the behavioral variables the PCA unveiled that the primary source of data variation is accounted for by the first three principal components, exceeding what would be expected from shuffled data (PC1: 15.01%; PC2: 14.84%; PC3: 12.21%; cumulative variance: 42.06%; Fig. 4A). Importantly, the first PCs discriminate between NH and H individuals in feature space (Fig. 4B). Additionally, they significantly differentiate between the NS and IS groups (PC2: t_(43)_ =  − 2.810, *p* = 0.007; PC3: t_(43)_ = 2.147, *p* = 0.037, Fig. 4C). Noteworthy, PC2 exhibits higher loadings on helplessness- and other susceptibility-related variables, such as escape failures (SB [Escape Failures], coeff. = 0.34) and time in closed arms of the EPM (EPM [Closed Arms], coeff. = 0.19), while exhibiting lower loadings on resilience-related variables, such as swimming in the FST (FST [Swim.], coeff. =  − 0.03) and sucrose preference (SPT [D1, Suc. Pref. %], coeff. =  − 0.33, Fig. 4D). Notably, PC1 and PC3 exhibit lower contributions from escape performance in the SB (PC1: coeff. =  − 0.21; PC2: coeff. =  − 0.21) and have opposing loadings of FST (e.g., FST [immob.]: PC1, coeff. = 0.39; PC3, coeff. =  − 0.18), indicating independent variability in both domains. Finally, we also fitted a linear discriminant model (LDM) on PC scores to predict helpless individuals. We observed better classification performances from the original data compared to shuffled data, with significant improvements by including PC2 (accuracy_(data)_ = 0.894, accuracy_(shuffled data)_ = 0.662, *p* = 0.007, LDA) and PC3 (accuracy_(data)_ = 0.907, accuracy_(shuffled data)_ = 0.656, *p* = 0.037, LDA, Fig. 4E). Collectively, these results demonstrate that most of the variation in the data is explained by the first components of the PCA, and these components capture the separation between NH and H individuals, as well as between susceptibility and resilience variables.

To evaluate the reliability of the multidimensional covariation patterns identified by PCA, we conducted correlations between the principal components (PCs) derived from the complete dataset and those from randomly partitioned data. Our analysis indicated that the PCs derived from the complete dataset consistently emerged in the partitioned data with reduced observations (Fig. S2A), but not in the shuffled data (Fig. S2B). Additionally, by selecting the most similar PC to the whole dataset PC for every iteration, we observed a consistently high correlation of the initial PCs, both for scores and coefficients, which did not occur in the shuffled data (e.g., PC1, median values of maximum iteration correlation: Coeff. = 0.856, Scores = 0.856; Coeff._(Shuffled)_ = 0.414, Scores_(Shuffled)_ = 0.412, Fig. S2C).

Subsequently, we conducted PCA using only one representative behavioral measure for each test. We opted for this approach due to the potential interdependence among variables within tests, with the objective of mitigating multicollinearity bias. Assessment through the multicollinearity test revealed that the chosen representative variables demonstrated minimal interdependence (VIF values < 1.1, Fig. S3A). PCA of these representative variables shows that the principal source of data variation is well explained by the first three principal components (PC1: 23.82%; PC2: 21.61%; PC3: 15.78%; cumulative variance: 61.21%; Fig. S3B). PC1 is primarily described by the covariation between reduced locomotion in the open field (OF [Total Distance], coeff. =  − 0.43), reduced immobility time in the FST (FST [Immob.], coeff. =  − 0.56), and increased sucrose preference (SPT [Average Pref.], coeff = 0.61). In the PC2, the main coefficients of covariation are reduced time spent in open arms of the EPM (EPM [Open Arms], coeff. =  − 0.42), reduced sucrose preference (SPT [Average Pref.], coeff =  − 0.32), increased social interaction (SIT [Soc. Pref. Ratio], coeff. = 0.53) and increased escape failures in the SB (SB [Escape Failures], coeff. = 0.61, Fig. S3D). In PC3, the principal source of variation is explained by increased time spent in open arms of the EPM (EPM [Open Arms], coeff. = 0.80) and escape failures in the shuttle box (SB [Escape Failures], coeff. = 0.57, Fig. S3D). Interestingly, PC1 is sensitive to the covariation between immobility time in the FST (moderately) and sucrose preference, two of the main measures of depressive-like behaviors. However, it is not sensitive to escape failures in the SB and does not discriminate animals between the groups (loading scores: IS vs. NS, t_(43)_ =  − 0.7881, *p* = 0.4349, Student's t-test; H vs. NH: t_(43)_ = 0.2943, *p* = 0.7698, Student's t-test, Fig. S3C). In contrast, PC2 and PC3 are described by high coefficients of escape failures in the SB, which covaries with social interaction in PC2 and time in open arms of the EPM in PC3, measures classically associated with depressive-like behavior and anxious-like behavior, respectively (Fig. S3D). Additionally, the scores of PC2 discriminate individuals between groups regardless of clustering, with IS and H groups being better described by positive scores, indicating high social interaction and high escape failures in the SB (loading scores: IS vs. NS, U = 662, *p* = 0.0026, Wilcoxon rank-sum test; H vs. NH: U = 795, *p* = 0.0004, Wilcoxon rank-sum test; Fig. S3C, E). In contrast, the scores of PC3 only discriminate individuals between H and NH groups, with the H group being mainly described by positive scores, indicating high time in open arms of the EPM and high escape failures in SB (loading scores: IS vs. NS, t_(43)_ = 1.8122, *p* = 0.0769, Student's t-test; H vs. NH: U = 817, *p* < 0.0001, Wilcoxon rank-sum test; Fig. S3C, E).

### Clustering of individuals discriminates stressed subjects and reveals multidimensional behavioral phenotypes of resilience and susceptibility

**Figure 5:**
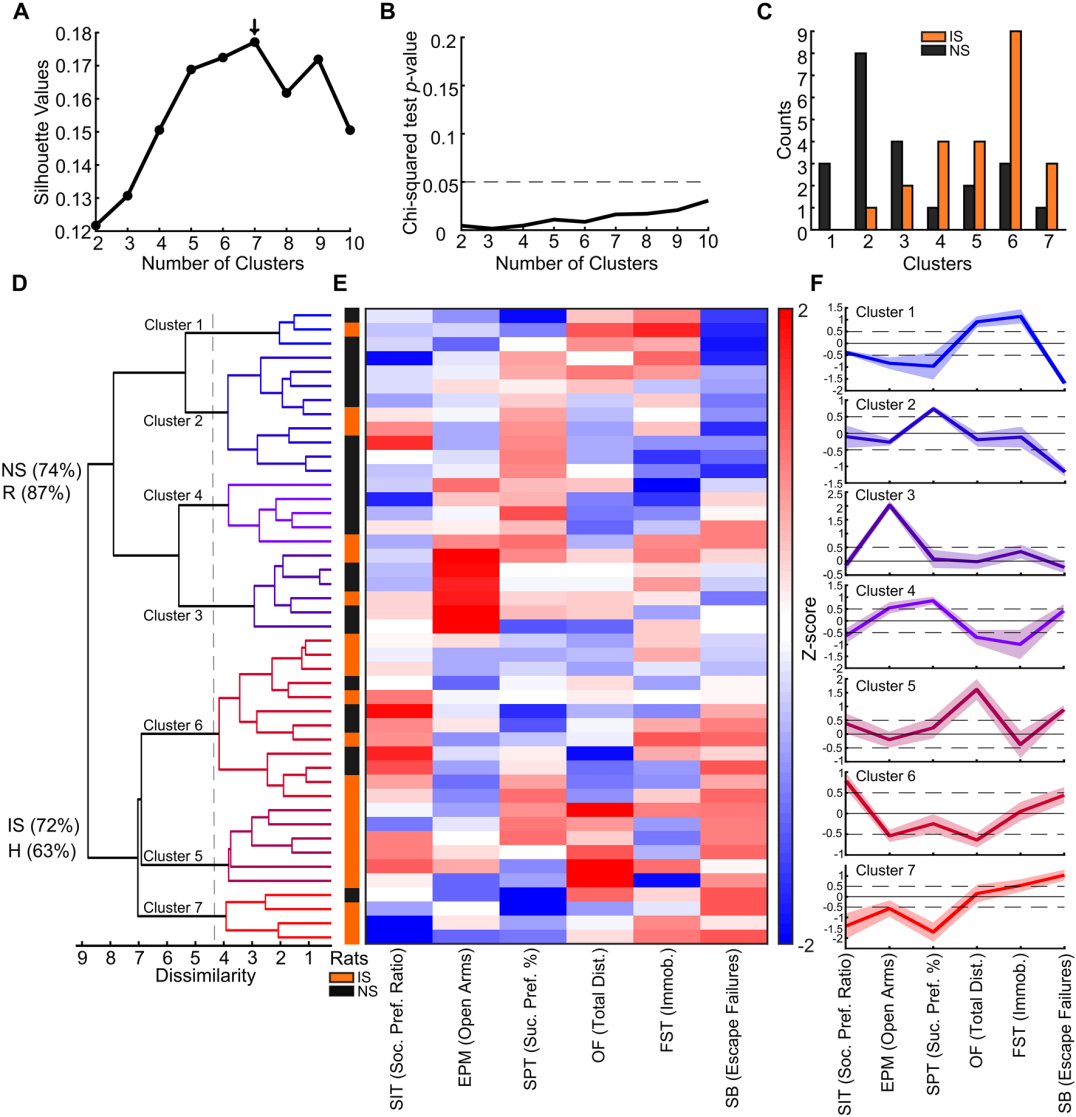
Clustering of individuals distinguishes stressed individuals and reveals multidimensional behavioral phenotypes of resilience and susceptibility. (**A**) Silhouette values indicate seven clusters as optimal for hierarchical clustering. (**B**) Every clustering hierarchy significantly (IS vs. NS Chi-squared test *p* value) distinguishes stressed individuals. (**C**) Clusters exhibit a spectrum of distinction between proportions of NS versus IS individuals. (**D**) Dendrogram of hierarchical clustering showing 7 clusters delimited at dissimilarity values of ~ 4.4 and (**E**) Z-scored data for each individual. Note that the two large hierarchical clusters (**D**; delimiting the dendrogram by dissimilarity values of ~ 8) show a clear distinction in SB escape performance (**E**). (**F**) Multidimensional behavioral profiles indicated by the different hierarchical clusters. Clusters show the Z-score value of each variable. Note that Cluster 7 presents a generalized profile of susceptibility. Also, note that Cluster 2 exhibits an overall profile associated with resilience. Lines and shaded boundaries represent the mean ± SEM.

Considering the remarkable diversity of multivariate patterns across behavioral variables described so far, as revealed by PCA, factor analysis, and feature clustering, we sought to investigate, using hierarchical clustering, whether this variability is related to the existence of distinctive clusters of individuals with similar behavioral profiles. The number of clusters was determined by evaluating the silhouette value, which indicated the formation of 7 clusters (Fig. 5A). The chi-squared test revealed significant discrimination of stressed individuals by every clustering hierarchy, i.e., from 2 to 7 clusters, (Fig. 5B) and demonstrated a significant difference in cluster proportions of individuals from IS and NS groups (e.g., 7 clusters × IS vs. NS: ${\text{X}}_{(6)}^{2}$ = 15.5632, *p* = 0.0163, Fig. 5C). The dendrogram of hierarchical clustering (HC) unveiled multivariate behavioral profiles of resilience and susceptibility (Cophenet coeff. = 0.4563, Fig. 5D). Notably, the dendrogram revealed two prominent clusters (dissimilarity ≈ 8) that distinctly delineate individuals based on their escape failures in the SB (Fig. 5E). Although most clusters present intermediate profiles, such as Clusters 1, 4, and 6, or are defined by specific variables, such as Clusters 3 (high time in the EPM open arms) and 5 (high OF total distance) (dissimilarity ~  = 4), generalized profiles of resilience or susceptibility are more prominently represented in Cluster 2 (resilience) and Cluster 7 (susceptibility, Fig. 5F). While the *resilience cluster* is primarily characterized by reduced escape failures in the SB associated with increased sucrose preference, strikingly, the *susceptibility cluster* stands out for displaying the most classic depressive-like behavioral alterations reported in the literature, such as reduced social interaction, reduced sucrose preference, increased immobility in the FST, and increased escape failures in the SB (Cluster 7, Fig. 5F).

Given that clustering algorithms can produce different results due to random and non-deterministic factors, it's crucial to verify the reliability of the outcomes yielded by the chosen clustering method. To ascertain whether various clustering algorithms expose comparable multidimensional behavioral patterns, we carried an additional unsupervised clustering analysis employing a non-hierarchical approach, specifically the k-means algorithm. Silhouette values indicated that six and seven clusters were suitable for clustering (e.g., 6 clusters: Silhouette value = 0.355, Fig. S4A), but only four to six clusters could discriminate NS versus IS (e.g., 6 clusters: *p* = 0.014, Fig. S4B). Therefore, we selected six clusters as the most suitable for stress-related clustering through this semi-supervised approach (Fig. S4C). As found before, the clusters showed a spectrum of distinction in proportions between NS versus IS individuals (${\text{X}}_{(5)}^{2}$ = 14.1991; *p* = 0.0144, Fig. S4D). Remarkably, the clusters identified by k-means exhibited a high correspondence with the hierarchical clusters, notably k-means Cluster 6, identical to HC Cluster 7, both characterized by a generalized susceptibility profile (Fig. S4E). This result demonstrates that in this dataset, two different clustering algorithms reveal similar multidimensional behavioral profiles.

## Discussion

Taken together, our results indicate that behavioral variables exhibit stronger correlations within tests than between tests and organize themselves based on their relation to susceptibility or resilience to stress. This variability translates into distinct multivariate behavioral profiles, including susceptibility or resilience profiles and intermediate phenotypes, indicating multiple stress coping styles.

Several studies have demonstrated that inescapable acute footshocks induce prolonged physiological and behavioral alterations (see Fig. S5/Supplementary Table 2). However, there is no consensus regarding the exact duration of these changes. In contrast to previous studies, our data do not confirm some frequently reported behavioral alterations that are observed over a prolonged period, such as reduced locomotion in the OF^38,39^ or time spent in the open arms of the EPM^39–41^. On the other hand, our data are consistent with a series of studies that do not observe alterations in measures such as immobility time in the FST^42^ or sucrose preference^39^. It is essential to note that despite the similarity of the protocols (a single session of acute stress), parameter differences, such as the number of trials and shock intensity, can yield heterogeneous experimental results. These differences may also be due to the time elapsed between stress and behavioral measurement. Short and Maier^43^ demonstrated that acute inescapable footshocks (100 trials, 1 mA) lead to a reduction in social interaction in the first 48 h, and this effect becomes non-significant three days after stress, possibly absent after seven days. This fact may explain why we did not observe alterations in the following tests, such as social preference and sucrose preference. However, it does not explain the changes observed in the startle response or in time in the open arms of the EPM.

In our study, a single session of inescapable footshock did not elicit group-level alterations in most of the evaluated behaviors. However, it was sufficient to induce learned helplessness and discriminate between H and NH individuals after 12 days. Interestingly, the induction of helplessness for 24 to 48 h by acute inescapable footshock is frequently reported^43–46, 47^. Still, the duration of this effect is not well-established, while some protocols report longer-lasting effects of the shock (e.g., 9 days in^48^). Notably, to our knowledge, our study is the first to show helplessness induction 12 days after acute inescapable footshock stress.

Our data revealed that covariation between reduced time spent in open arms of the EPM, reduced sucrose preference, and increased escape failures in the SB, all patterns captured by both all-variables PC2 and main-variables PC2, consistently distinguish IS from NS, as well as H from NH rats. This finding encompasses the main alterations typical of the depression-like profile reported in the literature. Indeed, various reports indicate the relationship between time in the open arms of the EPM and escape failures in SB. Naïve animals who spent more time in open arms exhibited reduced escape failures in the SB^49^, indicating an association between anxiety-like phenotype and helplessness. Similarly, rats selected for low sucrose consumption spend less time in the open arms of the EPM^50^, reinforcing the link between anhedonia and anxiety-like behavior. However, in the population of individuals in our study, the relationship between anxiety-like behavior and helplessness proved to be more complex. Our results show that increased time in the open arms of the EPM and increased escape failures, both captured by PC3, also discriminate H from NH rats, indicating, together with PC2, high variability in the relationship between stress susceptibility and different levels of anxiety-like behavior.

Importantly, our results revealed that the broad inter-individual behavioral variability captured by PCA translates into clusters of individuals with multidimensional behavioral profiles. Based on Koolhaas et al.^51^, these profiles found in our study can be interpreted as reflecting different stress coping styles, ranging from reactive to proactive coping and different emotional reactivity (e.g., hedonic preference). In general, some clusters are entirely represented by reactive or proactive coping styles, while others express both styles. This variation in coping styles throughout the battery of tests could indicate behavioral flexibility, with the subpopulations of individuals adapting differently to the demands of the tests^52^.

Interestingly, Kim et al.^53^ showed that mice subjected to a chronic protocol of inescapable shocks, regardless of susceptibility to stress, do not exhibit increased immobility time in the FST but exhibit anhedonia and anxiety-related alterations. Similarly, Stepanichev et al.^17^, using an eight-week chronic unpredictable mild stress or a two-week combined chronic stress protocol in rats, also did not observe changes in immobility time in the FST but reported stress-induced anhedonia. In fact, anhedonia is one of the core symptoms of depressive disorders and is often associated with helplessness in chronic footshock stress protocols. In contrast, Meng et al.^54^ demonstrated that one-week uncontrollable foot shocks only induced learned helplessness but not anhedonia in animals. However, our data show that the relationship between anhedonia and helplessness, in conjunction with other measures, explains a portion of the data variation (all-variables PC2 and main-variables PC2) and constitutes the susceptibility profile in a subset of individuals (HC Cluster 7, approximately 8.9% of the individuals in our study). Indeed, HC Cluster 7 is the only cluster that exhibits the major behavioral alterations most associated with the depressive-like phenotype. It is evident that the majority of depressive-like behavioral alterations only occur in a small portion of the population of rats, however, this proportion is intriguingly close to the prevalence of major depression in adults in the USA (8.8%^55^).

Notably, our data revealed that the immobility time in the FST, traditionally interpreted as a depressive-like alteration^56,57^, is not altered by inescapable footshocks. Furthermore, the immobility time shows weak correlations with other variables, and its relation with helplessness is unclear. Our data show that even individuals classified with high immobility time are unrelated to helplessness. Nevertheless, intriguingly, a subpopulation of resilient individuals (HC Cluster 1) exhibited high immobility time and a low number of escape failures, indicating that immobility may represent a possible adaptive stress-coping style as suggested previously^58–60^. Increasingly, studies have shown that immobility does not model “despair” or helplessness, nor does it reflect depression^58–61^. In fact, acute administration of glucocorticoids has been shown to increase immobility^62^, whereas the acute administration of glucocorticoid antagonists reduces immobility^63^. Both changes are directly related to hypothalamic–pituitary–adrenal axis activity and not exclusively to neurobiological aspects of depression. In this sense, high immobility time in the FST may represent a passive strategy for energy conservation, as well as reflecting aspects of learning and memory^64^. Similarly, in the SPT, the preference for water consumption over sucrose solution is interpreted as a reflex of anhedonia, one of the main symptoms of depression^65^, but the preference can sometimes be more related to other aspects of reward processing, such as motivation or impulse control, than to the inability to experience pleasure^66^.

Animal models of neuropsychiatric disorders aim to replicate aspects associated with clinical symptoms rather than reproduce the entire disorder’s characteristics^7^. In fact, after exposure to inescapable shock stress, only a few changes can be attributed to the development of depressive-like symptoms. Other observed alterations may have a greater significance in understanding aspects pertinent to other clinical conditions, such as anxiety and post-traumatic stress disorder (PTSD)^42,67^. Therefore, applying acute inescapable footshock stress protocols should not aim to model multifaceted conditions but rather specific symptoms that may reveal shared aspects among conditions.

In the clinical setting, even patients who meet the diagnostic criteria for major depression are characterized by differences in symptom profiles and severity levels, resulting in a myriad of heterogeneous clinical profiles^68,69^. For instance, according to the DSM-5 criteria, major depression diagnosis requires the presence of 5 out of 9 criteria, potentially resulting in two individuals sharing just 1 symptom yet receiving the same diagnosis^3^. This fact also points to individual biological variability as a relevant factor in the expression and relationship of depressive symptoms. Despite the majority of studies with stress-based animal models still neglecting individual variation^70^, an increasing number of studies have embraced this variable and provided essential insights^71–75^. In fact, our results demonstrate that inter-individual behavioral variability can reflect different stress-coping profiles, possibly in a similar way to the heterogeneity of symptoms observed in humans. In this sense, the multivariate assessment of individual behavioral responses can reveal patterns concealed by group mean values and highlight the implications of individual variability.

The use of dimensionality reduction, clustering, and classification techniques to investigate multidimensional behavioral profiles in behavioral test batteries is a promising but still underutilized strategy^27,31, 34–36, 76^. This approach has been applied in naive animals, allowing the characterization of inter-individual variability in different rodent strains^34–36^, and in animal models of stress-related disorders^27,31^, enabling reinterpretations of these models. Our results confirm patterns that are recurrent in these studies, such as (I) a higher correlation between measures of the same test than between tests^34,35^, indicating that measures of the same test may be capturing very similar alterations, (II) each principal component (PCA) or factor (factor analysis) explaining only a small portion of the data variance, typically between 20 and 30% of the variance in the first component or factor^34–36^, indicating that despite the existence of covariation patterns and phenotypic subpopulations, inter-individual variability is considerably high. Nonetheless, it is essential to note that in cases where the test battery measures few behavioral categories or the experimental subjects’ conditions are particular (e.g., a test battery with 3-week-old rats), the first component tends to capture a more significant portion of the data variation^27^.

Another relevant aspect is that distinct multidimensional patterns of behavioral alterations may indicate specific neurobiological mechanisms. For example, Ruggiero et al.^77^ examined various behavioral and neurobiological alterations following early-life seizure in rats and showed that a multivariate factor representing cognitive alterations (from the radial maze test) was more correlated with synaptic plasticity abnormalities, while a factor representing sensorimotor alterations (from the OF and PPI tests) was more correlated to neuroinflammation.

In our study, footshocks did not produce significant differences between the experimental groups in most of the behavioral tests used. However, the literature suggests that footshocks can induce behavioral changes when tests are used in a separate setting (Supplementary Table 2). Therefore, we should consider the possibility that the lack of between-group differences observed in our study is due to the experimental design adopted. The literature indicates that specific tests may impact the results of subsequent tests in the battery^78–80^. However, in this case, both experimental groups in our study would be equally exposed to these effects. Ideally, control groups should be included to assess the effects of footshocks separately for each test. However, using this type of experimental design would not enable the use of multivariate approaches, as it would require a very large sample size.

It is important to emphasize that other factors were controlled during the experimental battery, such as lighting that can provoke behavioral alterations^81^. The light intensity was adjusted in different protocols to minimize light as an aversive factor and avoid compromising performance, which could lead to anxiogenic behaviors described in the literature^82^, based on previously published protocols. Therefore, it is relevant to note that for tests such as the SIT^83^, EPM^84^, and NOR, lux levels were adjusted to a lower intensity. In contrast, the OF^85,86^, the FST^87,88^ and SPT were conducted under normal lighting conditions, while the PPI^89^ and SB were not conducted with lighting due to the apparatus configuration.

An important limitation of our study concerns the sample size adopted. Multivariate analyses require a large sample size to ensure a more stable assessment of the correlation coefficients between variables^90^. Although there is no consensus, some studies suggest that PCA and FA require a minimum sample size of 100^90,91^. However, it has been noted that a higher sample-to-variable ratio, meaning a sample size greater than the number of variables assessed, is crucial for obtaining quality results^90^. Although our study did not use a large sample size due to ethical reasons, we assumed a sample-to-variable ratio of 2:1 for all-variables analyses and a 4:1 ratio for main-variables analyses, obtaining similar results. Future studies using multivariate approaches should carefully plan sample size and sample/variable ratio to meet quality and ethical criteria for animal use.

A further important limitation of our study is the fact that only male rats were used. Studies using test batteries have reported behavioral differences between males and females^26,92^. Although a multivariate behavioral characterization of the effects of footshocks on animals of both sexes is beyond the scope of this study, future studies should consider including females in their experimental design. In addition, further studies should also consider evaluating multivariate behavioral profiles in test batteries after administration of classic drugs used in the management of depression to better understand the underlying mechanisms of stress-related depression.

Our results demonstrate that using a data-driven approach for the multidimensional assessment of behavioral profiles is a strategy that benefits from and makes sense of inter-individual variability, providing insights into the study of stress-related disorders, where understanding the basis of such variability is essential. Data-driven approach to clustering symptoms has helped to identify biomarkers, predict outcomes and improve treatment efficacy^6,93, 94^. This approach has gained increasing prominence in stress research and holds a significant position in the future of rodent models in depression research^14^.

## Conclusions

We have demonstrated that the multivariate assessment of behavioral responses to acute inescapable footshocks can reveal susceptibility or resilience behavioral profiles and intermediate phenotypes. Thus, our approach emphasizes the importance of considering individual variability and the relationship between multiple behavioral measures to understand different behavioral responses to stressors, providing new insights for the study of animal models of neuropsychiatric and stress-related disorders.

## Methods

### Animals

We utilized forty-eight male Wistar Hannover rats, aged seven to ten weeks old (weight range: 250–300 g), obtained from the Central Vivarium of the University of São Paulo, Ribeirão Preto Campus (USP-RP). Rats were housed under standard conditions in the housing room under a 12:12h light:dark cycle (lights on at 7:00 a.m.). In each home cage, four rats were conditioned to their respective experimental groups. The temperature was maintained at 24 ± 2 °C, and the rats had ad libitum access to food and water. Handling was carried out two days before the experimentation.

### Experimental design

Rats were randomly divided into a 'no shock' group (NS; not exposed to stress before the test battery; n = 24) and an 'inescapable shock' group (IS; exposed to inescapable electric footshocks before the test battery; n = 24). Subsequently, the rats were subjected to a comprehensive behavioral battery test on consecutive days. The home cages were transported from the housing room to the testing room on a cart with rubber wheels. After transport, the animals were kept in an anteroom for 1 h for acclimatization to reduce stress caused by transport and were kept in the testing room for 20 min before each test started. Lighting in the rooms was kept constant during all procedures. All apparatus was cleaned with an alcohol solution (variable concentration, between 30 to 70%, depending on the test performed) and deionized water. Experiments were carried out in the light cycle, 12:00 a.m. to 6:00 p.m., except for inescapable shock sessions starting at 7:00 a.m. and ending at 6:00 p.m. due to the duration of each session (1 h and 20 min per animal). Three animals were excluded from the study due to health-related issues (N = 1) or to technical issues (N = 2; final sample: NS = 22; IS = 23). All behavioral tests were videotaped using a Logitech C270 Webcam. In addition, we employed a Standard Multilaser Night Vision webcam adapted to the red and low light conditions required by the SIT protocol. The camera's infrared filter was removed, enabling it to capture both infrared and visible light, ensuring adequate video quality under these conditions. The behaviors in the FST, EPM, SIT, and NOR tests were quantified using the X-Plo-Rat software (X-Plo-Rat version 2005 1.1.0, https://github.com/lanec-unifesspa/x-plo-rat^95,96^), while the behaviors in the OF, PPI, and SB tests were automatically quantified using the software linked to each apparatus, providing pre-quantified measures. The measurements in the SPT were obtained analogically, with solutions being measured using a graduated cylinder and weighed with a digital scale. The recorded video files were named according to the animal's identification number, experimental group, date, and acquisition time, saved as .mp4 files, and stored on an external hard drive. All data were stored in compliance with ethical and privacy standards to protect the information contained in the data. In the tests that required video analysis, the identification of the rats and their experimental group was hidden to allow the analysis to be performed blindly by two researchers. All procedures were performed according to the ARRIVE guidelines and the National Council for the Control of Animal Experimentation guidelines for animal research. The study also was approved by the local Ethics Committee on the Use of Animals (Ribeirão Preto Medical School, University of São Paulo; protocol number: 78/2020).

### Terminology

In this article, we will adopt specific terminology to consistently refer to the diverse multivariate representations and data categories. We will use behavioral *dimension*, *variable*, and *feature* interchangeably to refer to a measurement or count that varies across observations ranging from low to high values. In our study, o*bservations* refer to the individuals (i.e. rats). Additionally, we may also use the term “feature” to describe the state of a variable (e.g., low latency to escape is a resilience-related feature). Of note, *feature clustering* is synonymous with the clustering of variables, while *individual clustering* is with the clustering of observations.

We use *multidimensional* and *multivariate* interchangeably to refer to analyses, patterns, and profiles that consider multiple variables simultaneously. Conversely, *univariate* refers to analyses where only a single variable is examined (e.g., comparing latency to escape between groups).

The term *patterns* refers to instances of multivariate relationships expressed through linear combinations of weighted coefficients (e.g., principal components’ coefficients and factor loadings) or the strength of associations between variables (e.g. feature clustering and correlation matrix).

In turn, *profiles* represent groupings of observations distinguished by unique combinations of multiple variables’ states (e.g., one profile may be characterized by high locomotion, low social interaction, and high latency to escape). Profiles can be revealed by clustering algorithms (e.g. hierarchical clustering or *k*-means) or by pronounced scores of multivariate patterns (e.g., principal component scores). When referring to clusters formed through unsupervised machine learning techniques, we commonly use the term “natural” clusters.

We designate as *resilience-related variables* those increased as responses to antidepressants or anxiolytics or those decreased in animal models of depression and anxiety. Conversely, *susceptibility-related variables* are characterized by higher values indicating a more pronounced impact of stress exposure in animal models or those intended to be reduced through pharmacological treatments. Additionally, certain variables are indices or ratios. In such cases, if the value is resilience-related, then the denominator value of the ratio is classified as susceptibility-related, and vice versa. These classifications are based on the established validity of the models used in our study. The behavioral variables, their respective classifications, and the literature that based them are provided in Supplementary Table 1.

Across investigations, we will use *within-test* and *intra-test* interchangeably when examining distinct variables presented in one session (e.g., FST train) or the same test in different sessions (e.g., FST train and FST test; SPT days 1, 2, and 3). Examinations of variables from different tests will be denoted as *between-tests* or *inter-tests*.

### Behavioral test battery

The test battery was composed of tests classically used in behavioral neurosciences to investigate psychiatric-like behavioral alterations related to stress. Tests were performed on consecutive days, according to the following sequential order: OF, FST, SIT, EPM, NOR, PPI/startle, SPT, and SB. The most potentially stressful tests were intercalated with the least stressful tests, thus avoiding the concentration of more stressful tests in a short time.

### Inescapable footshocks

Rats were exposed to inescapable footshocks in an adapted active avoidance shuttle box apparatus (Insight Ltda^12^). The apparatus consists of a floor with metal bars, LEDs for emitting light stimulus, eight infrared beams to detect animal location, and a shock generator with an electric current limiter. The apparatus has dimensions of 30.7 cm in height × 33 cm in width × 54 cm in length and was placed in a sound-attenuating chamber acoustic insulation box (51 cm in height × 48 cm in width × 63 cm in diameter).

The IS group was exposed to 60 trials of electric foot shocks. Each trial started with exposure to a luminous stimulus (200 lx; conditioned stimulus) for 20 s. Ten seconds after the stimulus started, the animals received inescapable shocks with an intensity of 0.8 mA and an uninterrupted duration of 10 s. Both stimuli ended simultaneously and were followed by a random 40 ± 20 s interval between trials. The NS group was submitted to the same protocol but received no shocks. The entire experiment was carried out in a dark room (0 lx^12^).

### Open field test (OF)

The OF is used to assess the locomotion and anxiety-like behavior of animals when exposed to an open environment^85,97, 98^. The apparatus consists of a white floor arena (Insight Ltda; 46 cm in height and 46 cm in length) surrounded by translucent walls made of plexiglass. In this test, the animals were placed individually in the arena center to explore the environment freely for 20 min. Throughout the task, the lighting intensity in the room was kept constant (500 lx^85,86^). The movements were captured by infrared sensors detecting presence and movement arranged on the walls of the arena. For the analyses, we considered the distance traveled, the time in the center, and the rearing events as measures related to anxiety-like changes^39,85, 97–100^. Distance traveled was quantified across 5-min blocks.

### Forced swim test (FST)

The FST has been discussed as evidence of “behavioral despair” and is commonly used to assess depressive-like behaviors and antidepressant responses. Longer immobility time and shorter escape-oriented behaviors are measures associated with the depressive-like phenotype^101^. In this study, we carried out the test in two stages, each in a day, under the same environmental conditions and constant lighting of 500 lx^87,88^. On the first day, the animals were placed in the center of a transparent acrylic cylinder (60 cm in height and 30 cm in length, filled with water up to 30 cm in height at 25 °C) for 15 min. Twenty-four hours later, the animals were again subjected to the task in the same settings for 5 min. After each session, the rats were carefully dried before returning to their cages. For the analyses, we considered immobility time, climbing time and swimming time as measures related to depressive-like changes^56,101^.

### Social interaction test (SIT)

The SIT evaluates socially motivated behaviors represented by the approach or avoidance (discussed as “social anxiety”) between the animals tested^102^. In our study, the test was adapted from Lukas et al.^103^ and carried out in the previously described open field. Rats were initially habituated to the apparatus for 30 s and then to the presence of an empty cage (12.5 cm in height and 9 cm in width) positioned against a wall of the arena for 5 min. Subsequently, the tested rat was removed from the arena for 2 min to accommodate the conspecific animal (same strain, sex, age, size, and weight) in the cage. The tested animal was reinserted in the arena for 10 min, and all of its movements were videotaped with an infrared camera. The experiment was carried out under constant red lighting with other lights turned off (7 lx^83^). For the analyses, we considered time spent interacting with the empty cage or the cage occupied by the conspecific, and time in corners as measures related to anxiety-like changes^67,103–105^. The interaction behavior was determined as the time in which the tested animal head was directed to the cage, and its nose was at a distance of approximately 1 cm or less. We calculated the social preference ratio as OC/EC, where OC = time spent interacting with the occupied cage and EC = time spent interacting with the empty cage^105^. Time in corners was calculated as OC/EC, where OC = time in corners during occupied cage and EC = time in corners during empty cage^105^.

### Elevated plus maze test (EPM)

The EPM assesses behaviors associated with innate anxiety^106^. The test generates a conflict between the environmental novelty exploration drive and the innate fear of open places in rodents. More time spent in closed arms and avoiding open arms are associated with the anxious-like phenotype. The behavioral apparatus (80 cm in height, 110 cm in width, and 110 cm in length) consists of four elevated arms of the same size intersecting at a central point. Two arms are closed by side walls and the other two are open. The apparatus is 30 cm away from the room walls, in an environment without visual, olfactory, or auditory clues, and under constant exposure to homogeneous lighting (2 lx^84^). In this test, animals were placed in the center of the apparatus and videotaped with an infrared camera for 5 min. For the analyses, we considered the time spent in the open arms and in the closed arms, and the risk assessment as measures related to anxiety-like changes^39,103, 104, 106, 107^. The risk assessment was defined as the posture of stretching in the direction of the open arms of the EPM.

### Novel object recognition test (NOR)

The NOR assesses the recognition memory based on the exposure of a familiar object maintained throughout the sessions and a new object that varies between each session. A low discrimination ratio of the new object compared to the familiar object is associated with cognitive deficits^108^. In this study, the task was performed in a closed arena (83 cm in height × 42.5 cm in width × 39.5 cm in length), with its lighting (150 lx) and an air exchange system (fan attached to the arena wall) over three days. On the first day, animals were habituated to the apparatus for 20 min. Twenty-four hours later, the habituation lasted 10 min and was followed by exposure to two identical objects (objects A and A′) inserted in the arena for 5 min. After a 15-min interval outside the arena, the animal was reinserted in the apparatus for 5 min and exposed to the objects (A, familiar object, and B, new object). In this stage, we tested the animal's short-term memory based on the discrimination between objects A and B. The next day, the reexposure to objects (A, familiar object, and C, new object) lasted 5 min. At this stage, we tested the animal's long-term memory and discrimination between familiar and new objects. The entire experiment was carried out under constant lighting and was videotaped. For the analyses, we considered the relative and the total time of object interaction as measures related to cognitive changes^108^. We calculated the new object discrimination index (short-term trial and long-term trial) according to the formula: TN/(TF + TN), where TN = new object exploration time and TF = familiar object exploration time.

### Prepulse inhibition and acoustic startle reflex test (PPI/startle)

The PPI/Startle assesses the startle motor response caused by an intense sound stimulus (pulse). This response is reduced when the pulse is preceded by a less intense sound stimulus, the prepulse^109^. In this test, the animals were exposed to trials of pulses prepulse, prepulse + pulse, and were only evaluated for their startle response or inhibition. The sound stimuli were generated by two loudspeakers located inside an acoustic isolation box. The animals were restrained to a cage (12.5 cm in height, 9 cm in width, and 19 cm in length) coupled to a stabilometer that measures the motor reaction. After the initial 5 min of habituation to the apparatus, the animals were subjected to 60 semi-randomized sound stimulus trials between pulse stimuli (6 stimuli; 120 dB intensity; 40 ms duration), 18 prepulse stimuli (71, 77, and 83 dB intensity; 6 stimuli for each intensity; 20 ms duration), 18 prepulse + pulse stimuli (prepulse at 71, 77, and 83 dB intensity; 6 stimuli for each intensity; 20 ms duration, followed after 100 ms by the pulse of 120 dB of intensity and 40 ms of duration) and only background noise stimulus (6 trials; 65 dB of intensity). The interval between each trial lasted 15 ± 8 s (adapted from^77^). The lighting of the room (500 lx) and from inside apparatus (0 lx^89^) remained constant during the experiment. The following behavioral measures recorded by the software integrated into the apparatus (Insight Ltda) were considered for analysis: the pulse trials mean (represented as startle reflex amplitude), the startle habituation (HAB) percentage (%HAB = 100 − (100*(HAB1/HAB0))), and the prepulse inhibition percentage for three stimulus intensities (%PPI = 100 − (((PP + P)/P)*100)).

### Sucrose preference test (SPT)

The SPT is traditionally used as an indicator of anhedonia, one of the main symptoms of depression^66^. The absence of preference for sucrose compared to water is associated with anhedonia^110^. In this test, animals were housed individually in home cages for 72 h with ad libitum access to two drinking fountains: one containing only water and the other containing 1% concentrated sucrose solution, both filled with 500 ml. Every 24 h, the volume of liquid remaining was measured, and the drinking fountains were switched positions (left vs. right) in the home cages. The animals were handled only for weighing and there was no additional exposure to light. For the analyses, we considered daily water and sucrose intake, and daily and total sucrose preference as measures related to depressive-like changes^17,66, 110^. Daily sucrose preference was calculated as (SI/(SI + WI))*100, where SI = sucrose intake and WI = water intake^110^. Total sucrose preference was calculated as the average of daily sucrose preference.

### Shuttle box (SB)

The SB with escapable footshocks evaluates the escape response to controllable stress. In this test, the shock duration is controllable, in contrast to the inescapable shocks that are fixed^11^. For the shock termination, animals must jump over a 1-cm high wall obstacle (an escape response) placed in the shuttle box center until the 10-s maximum duration. The high number of escape failures and mean escape latencies are interpreted as “learned helplessness” indicators. In our study, the task consisted of 30 escapable shock trials with 0.6 mA intensity. Each trial consisted of a light stimulus lasting 10 s, followed by a light stimulus + shock lasting 10 s, and, finally, an interval of 40 ± 20 s without stimuli. The 5 first trials had a fixed ratio-1 (FR-1) task acquisition criterion, in which shock termination was conditioned to the execution of a single escape response performed during the shock duration interval (10 s). The subsequent 25 trials had a fixed ratio-2 (FR-2) in which the termination of shock was conditioned to two escape responses. The experimental room remained without lighting (0 lx^12^), except for the sporadic luminosity of the apparatus' light stimulus (200 lx), and animal movements were videotaped with an infrared camera. For the analyses, we considered FR-1 mean escape latencies, FR-2 mean escape latencies, and the number of escape failures as measures related to depressive-like changes^12,111,112^. All measures were obtained by the apparatus-integrated software (Insight Ltda). Escape latencies are defined as the time interval between the shock onset and each escape performed.

### Statistical analysis

We used the Kolmogorov–Smirnov test to infer a normal distribution between experimental groups. We used the Student's *t*-test to compare samples following a normal distribution and the Wilcoxon rank-sum test as a non-parametric alternative. To assess the equality of variances between the experimental groups we used the Levene Test for Equality of Variances. These procedures were performed using custom MATLAB (R2022b, Mathworks^113^) code. Data are represented as the mean ± standard error of the mean (SEM) for line and bar plots. Box-plot data are represented as median and interquartile range, with whiskers as range.

### Reliability analysis

We performed reliability analyses on behavioral measures requiring manual quantifications, namely FST, EPM, NOR, and SIT. Reliability was assessed by computing Cohen’s kappa, Pearson’s correlation, and the intraclass correlation coefficient (ICC) of absolute agreement between measures^114^. To ensure the X-Plo Rat software reliability, we conducted re-quantifications using BORIS (Behavioral Observation Research Interactive Software) —another free open-source behavioral analysis software—in a randomly selected subset of subjects (N = 5). We obtained a strong correlation between measurements (r = 0.9392) and an ICC of 0.9244, demonstrating an excellent equivalency between the X-Plo Rat and BORIS measurements. To ensure inter-rater reliability of the reference rater, we conducted re-quantifications by two raters using BORIS in a randomly selected subset of subjects (N = 5). Cohen’s kappa obtained throughout second-by-second agreement of occurrences of behavioral events revealed a substantial agreement in the range of averages across variables (0.65–0.81). Moreover, the quantifications between the two raters revealed a strong correlation (r = 0.9805), ranging from 0.9187 to 0.9978 across variables. Finally, we observed an excellent inter-rater ICC of 0.9783, demonstrating adequate inter-rater reliability.

### Multivariate analysis

Our multivariate analysis primarily focused on investigating the relationships between data variables across different behavioral tests. The aim was to uncover patterns that could reveal distinct phenotypes characterized by specific behavioral traits or factors that might not be evident when examining population-level averages alone or pairwise correlations. To determine the most relevant behavioral variables for each test, we considered theoretical principles, incorporating classical variables from the literature, and encompassing various behavioral categories, such as anxiety measures, recognition memory, sociability, anhedonia, sensorimotor gating, helplessness, and exploration.

All multivariate analysis was performed in MATLAB using custom codes and built-in algorithms. Initially, all variables of each animal were normalized by subtracting the mean of the samples from both groups and dividing by the standard deviation of the mean (Z-score) for each behavioral variable.

### Linear correlation

We used Spearman's correlation to identify correlation between variables, obtaining the *p* value and the correlation coefficient (r_s_).

### Principal component analysis (PCA)

PCA is a dimensionality reduction method that summarizes multivariate data into a smaller set of variables called principal components (PC). Each PC is a linear combination of the original variables with weighted *coefficients* at each variable. These components can then be projected onto the original data (sum of pointwise multiplication) giving each PC *scores*. To obtain these components the original data's multiple dimensions (in our study, different behavioral variables) are rotated to align with new axes that capture the greatest variance in the dataset. These axes are orthogonal, ensuring that there is no correlation between each other and thus reveal distinct covariance patterns within the multidimensional data with no redundancy. In the end, the number of PCs is the same as the number of variables. However, because they are ranked by the amount of variance explained, fewer PCs already capture most of the data variance, especially in multivariate data that exhibits associations within. Thus, these components represent the underlying structure of the data and can be used to identify patterns, relationships, and outliers. In our study, we performed PCA using the MATLAB *pca* function (https://www.mathworks.com/help/stats/pca.html) using the singular value decomposition algorithm, widely used for PCA due to its flexibility to data structure and robustness to noise and redundancy. The basic algorithm steps include data standardization, computation of the covariance matrix, singular value decomposition, selection of principal components, and reconstruction of the data by projecting the selected components.

### Factor analysis

Factor analysis is also a dimensionality reduction method that uncovers latent factors from data based on multidimensional correlations. Differently from PCA, which aims to identify axes that capture the maximum variance in the data, factor analysis seeks to uncover latent factors that explain the correlations between variables. Such *factors* are also composed of linear combinations of weighted coefficients at each variable called *loadings* and can be interpreted as representing specific behavioral constructs. In this analysis, we are allowed to define the number of factors to discover and the factor rotation method, which can influence the comprehensiveness of variables gathered into each factor. In our study, we performed common factor analysis using the MATLAB *factoran* function (https://www.mathworks.com/help/stats/factoran.html) using varimax rotation. Varimax rotation is commonly used in factor analysis. The goal of varimax rotation is to maximize the variance of the squared loadings within each factor, leading to simpler and more interpretable factor structures. By maximizing the variance of the loadings, varimax rotation tends to produce factors with high loadings on a small number of variables, making it easier to interpret the relationship between variables and factors, which was particularly suitable for our investigation of intra-test versus inter-test behavioral relationships. To define a relevant and interpretable number of factors to apply in our study, we previously inspected factor loadings for different predefined numbers of factors ranging from 2 to 11.

### Linear discriminant model (LDM)

In addition to our predominant approach of employing *unsupervised* analysis to discover natural patterns and profiles, that is, without considering group labels, we also conducted a *supervised* approach, where we examined how the multivariate data could classify individuals with taught behavioral categories. To assess how the collective multivariate patterns discriminate H from NH individuals, we fitted a regularized linear discriminant analysis classifier on the cumulative sets of PCs’ scores (PC1 to PC2, PC1 to PC3, PC1 to PC4, and so on). We estimated the average accuracy across a thousand iterations where we trained the model with randomly selected 70% of observations and tested on the remaining 30%. For that, we used the MATLAB *fitcdiscr* function (https://www.mathworks.com/help/stats/fitcdiscr.html). We used the linear discriminant type because it is the most easily interpretable. To promote the least transformation of the data, we chose linear coefficient threshold (delta) as zero and no gamma regularization as parameters.

### Clustering analysis

Clustering analysis is used to group variables (e.g., behaviors) or observations (e.g., rats) without previously defining classes (unsupervised). These algorithms try to find similar data and merge them into a single cluster. In this way, data presenting more similar features are grouped into a class. In our study, we conducted clustering analysis on observations (individuals) to assess the formation of multivariate behavioral profiles and on variables (behaviors) to evaluate the relationship between behavioral measures.

We employed agglomerative hierarchical tree clustering algorithms, which establish a hierarchical relationship among the analyzed data by connecting closer data pairs based on a distance metric. These clusters are subsequently merged into larger clusters, and the distances between clusters and all other points are calculated, with the closest cluster pairs continuously connected. The linear correlation coefficient between the cophenetic distances derived from the hierarchical tree and the original distances (or dissimilarities) used to construct the tree determines the cophenetic correlation, which characterizes the hierarchical relationships among clusters. In our study, we performed hierarchical clustering with the MATLAB *linkage* function (https://www.mathworks.com/help/stats/linkage.html). To calculate the cophenetic correlation, we used the MATLAB *cophenet* function. We used Euclidean distance between points as a distance metric and Ward's method of minimum variance to establish the link between clusters. Euclidean distance is a measure of the straight-line distance between two points in space, defined as the square root of the sum of the squared differences between the coordinates of corresponding dimensions of the two points. This is an appropriate measure for hierarchical clustering because it is simply implemented, easily interpretable, sensitive to magnitude, robust against outliers, appropriately handles non-linearity, and is the recommended metric for Ward’s method. Here, the distances between points and nodes are denoted *dissimilarity*. We calculated Euclidean distance with MATLAB’s *pdist* and *pdist2* functions. In Ward's method, at each step of the clustering process, the pair of clusters that leads to the smallest increase in total within-cluster variance when merged is selected. This means that the clusters are combined in a way that minimizes the overall dispersion within each cluster. Ward's method is advantageous because it tends to produce compact, well-separated clusters. It is particularly useful when the goal is to identify clusters with relatively low within-cluster variance.

In our investigation, we also intended to perform hierarchical clustering aiming to link multiple variables that exhibit either positive or negative relationships. However, the Euclidean distance metric, despite its advantages, only links variables with a positive relationship. Consider the following scenarios: In the first scenario, variable A demonstrates high values when variables B and C are also high, and low values when B and C are low, indicating a pattern of covariation among A, B, and C. Conversely, in the second scenario, variable A is high when variables B and C are low, and low when B and C are high, indicating another pattern of covariation despite the negative associations between A and B, C. In our dataset, where variables were Z-scored, the distributions of B and C values are very similar to the negative values of A. Therefore, to account for the strength of association regardless of its sign—positive or negative—we devised a modified “sign-independent” Euclidean distance metric. For that, we calculated the Euclidean distance of one point to the other, then we calculated it again but with the negative values of the other, and subsequently chose the minimum of these two values.

We also employed k-means clustering of individuals for two purposes: (I) to classify H versus NH rats based on the number of escape failures and mean latency to escape and (II) as a second alternative of clustering algorithm to compare the multivariate profiles obtained with hierarchical clustering. The k-means clustering method separates data into a predefined number of clusters using a distance metric between the data and centroids, with centroids initially established randomly or predefined, and iteratively recalculated by taking the mean of associated points until convergence is achieved. We performed k-means clustering using the MATLAB *kmeans* function (https://www.mathworks.com/help/stats/kmeans.html). For this method, we used the squared Euclidean distance as a measure, as well as random values of the initial centroid. The choice of the cluster number was based on silhouette values (a measure of the proximity to other data in the cluster in relation to the data closest to a neighboring cluster). We repeated the clustering method ten thousand times because the initial centroid values are random, so the convergence can occur in local minimums, leading to possible variability between replicates, and we chose the result that presented the lowest intra-cluster variance.

Finally, to evaluate how natural clusters separated experimentally defined behavioral classes, we performed Chi-squared tests of clusters × classes. For variables, we tested how clusters differentiated resilience-related from susceptibility-related variables. Also, we tested how clusters differentiated variables from different tests. For observations, we examined how clusters separated IS from NS individuals.

### Multicollinearity analysis

Considering that within-test variables may be interdependent and aiming to avoid multicollinearity bias in the data, we also carried out the analyses with behavioral measures representative of each behavioral test, opting for measures commonly used in the evaluation of neuropsychiatric disorder models (locomotion in the OF; immobility in the FST; social interaction; time in open arms of the EPM; sucrose preference; and escape failures in the SB). Multicollinearity between selected measures was assessed using the Variance Inflation Factor (VIF) method, which identifies the correlation between variables and the strength of this correlation, indicating the degree of interdependence of these variables. VIF values above 1.1 were assumed to indicate multicollinearity.

### Supplementary Information

Supplementary Information.

## Data Availability

All data have been deposited at https://osf.io/axynb and are publicly available.

## Code availability

The code for the analyses presented in this paper is openly accessible at https://github.com/rafaruggiero/Oliveira-Junior-SciRep2024.

## References

[1] World Health Organization (2017). Depression and Other Common Mental Disorders: Global Health Estimates.

[2] Greenberg PE, Fournier AA, Sisitsky T, Pike CT, Kessler RC (2015). The economic burden of adults with major depressive disorder in the United States (2005 and 2010). J. Clin. Psychiatry.

[3] Kendler KS (2016). The phenomenology of major depression and the representativeness and nature of DSM criteria. Am. J. Psychiatry.

[4] Blackburn TP (2019). Depressive disorders: Treatment failures and poor prognosis over the last 50 years. Pharmacol. Res. Perspect..

[5] Williams LM (2016). Precision psychiatry: A neural circuit taxonomy for depression and anxiety. Lancet Psychiatry.

[6] Drysdale AT (2017). Resting-state connectivity biomarkers define neurophysiological subtypes of depression. Nat. Med..

[7] Nestler EJ, Hyman SE (2010). Animal models of neuropsychiatric disorders. Nat. Neurosci..

[8] Charney DS, Manji HK (2004). Life stress, genes, and depression: multiple pathways lead to increased risk and new opportunities for intervention. Sci. STKE..

[9] Alfonso J, Frasch AC, Flugge G (2005). Chronic stress, depression and antidepressants: Effects on gene transcription in the hippocampus. Rev. Neurosci..

[10] Leigh B, Milgrom J (2008). Risk factors for antenatal depression, postnatal depression and parenting stress. BMC Psychiatry.

[11] Maier SF, Seligman ME (2016). Learned helplessness at fifty: Insights from neuroscience. Psychol. Rev..

[12] Marques DB, Ruggiero RN, Bueno-Junior LS, Rossignoli MT, Leite JP (2022). Prediction of learned resistance or helplessness by hippocampal-prefrontal cortical network activity during stress. J. Neurosci..

[13] Insel T, Cuthbert B, Garvey M, Heinssen R, Pine DS, Quinn K, Wang P (2010). Research domain criteria (RDoC): Toward a new classification framework for research on mental disorders. Am. J. Psychiatry..

[14] Gururajan A, Reif A, Cryan JF, Slattery DA (2019). The future of rodent models in depression research. Nat. Rev. Neurosci..

[15] Bhatnagar S (2021). Rethinking stress resilience. Trends Neurosci..

[16] Strekalova T, Spanagel R, Bartsch D, Henn FA, Gass P (2004). Stress-induced anhedonia in mice is associated with deficits in forced swimming and exploration. Neuropsychopharmacology.

[17] Stepanichev MY (2016). Anhedonia but not passive floating is an indicator of depressive-like behavior in two chronic stress paradigms. Acta Neurobiol. Exp..

[18] Kompagne H, Bárdos G, Szénási G, Gacsályi I, Hársing LG, Lévay G (2008). Chronic mild stress generates clear depressive but ambiguous anxiety-like behaviour in rats. Behav. Brain Res..

[19] Brown PL, Hurley C, Repucci N, Drugan RC (2001). Behavioral analysis of stress controllability effects in a new swim stress paradigm. Pharmacol. Biochem. Behav..

[20] Morgan BJT, Simpson MJA, Hanby JP, Hall-Craggs J (1976). Visualizing interaction and sequential data in animal behaviour: Theory and application of cluster-analysis methods. Behaviour..

[21] Huntingford FA (1976). An investigation of the territorial behaviour of the three-spined stickleback (Gasterosteus aculeatus) using principal components analysis. Anim. Behav..

[22] Spruijt BM, Gispen WH (1984). Behavioral sequences as an easily quantifiable parameter in experimental studies. Physiol. Behav..

[23] Espejo EF, Mir D (1993). Structure of the rat's behaviour in the hot plate test. Behav. Brain Res..

[24] Casarrubea M, Sorbera F, Crescimanno G (2009). Multivariate data handling in the study of rat behavior: An integrated approach. Behav. Res. Methods..

[25] Lister RG (1987). The use of a plus-maze to measure anxiety in the mouse. Psychopharmacology.

[26] Ramos A, Berton O, Mormède P, Chaouloff F (1997). A multiple-test study of anxiety-related behaviours in six inbred rat strains. Behav. Brain Res..

[27] Sáenz JCB, Villagra OR, Trías JF (2006). Factor analysis of forced swimming test, sucrose preference test and open field test on enriched, social and isolated reared rats. Behav. Brain Res..

[28] Cohen H, Zohar J, Matar MA, Kaplan Z, Geva AB (2005). Unsupervised fuzzy clustering analysis supports behavioral cutoff criteria in an animal model of posttraumatic stress disorder. Biol. Psychiatry.

[29] Espejo EF (1997). Structure of the mouse behaviour on the elevated plus-maze test of anxiety. Behav. Brain Res..

[30] Casarrubea M, Jonsson GK, Faulisi F, Sorbera F, Di Giovanni G, Benigno A, Magnusson MS (2015). T-pattern analysis for the study of temporal structure of animal and human behavior: A comprehensive review. J. Neurosci. Methods.

[31] Kanari K, Kikusui T, Takeuchi Y, Mori Y (2005). Multidimensional structure of anxiety-related behavior in early-weaned rats. Behav. Brain Res..

[32] Lorsch ZS, Ambesi-Impiombato A, Zenowich R, Morganstern I, Leahy E, Bansal M, Hanania T (2021). Computational analysis of multidimensional behavioral alterations after chronic social defeat stress. Biol. Psychiatry.

[33] Peters SM, Pothuizen HH, Spruijt BM (2015). Ethological concepts enhance the translational value of animal models. Eur. J. Pharmacol..

[34] Feyissa DD (2017). Individual differences in male rats in a behavioral test battery: A multivariate statistical approach. Front. Behav. Neurosci..

[35] Kassai F, Ernyey AJ, Plangár I, Gyertyán I (2022). Lack of general learning ability factor in a rat test battery measuring a wide spectrum of cognitive domains. J. Integr. Neurosci..

[36] Rudolfová V (2022). Inter-individual differences in laboratory rats as revealed by three behavioural tasks. Sci. Rep..

[37] Inkscape Project. Inkscape. Retrieved from https://inkscape.org (2020).

[38] Van Dijken HH, Van Der Heyden JA, Mos J, Tilders FJ (1992). Inescapable footshocks induce progressive and long-lasting behavioural changes in male rats. Physiol. Behav..

[39] Kinn Rød AM (2012). Long-term effects of footshock and social defeat on anxiety-like behaviours in rats: Relationships to pre-stressor plasma corticosterone concentration. Stress.

[40] Steenbergen HL, Farabollini F, Heinsbroek RPW (1991). Sex-dependent effects of aversive stimulation on holeboard and elevated plus-maze behavior. Behav. Brain Res..

[41] Steenbergen HL, Heinsbroek RP, Van Hest A, Van de Poll NE (1990). Sex-dependent effects of inescapable shock administration on shuttlebox-escape performance and elevated plus-maze behavior. Physiol. Behav..

[42] Ronzoni G, Del Arco A, Mora F, Segovia G (2016). Enhanced noradrenergic activity in the amygdala contributes to hyperarousal in an animal model of PTSD. Psychoneuroendocrinology.

[43] Short KR, Maier SF (1993). Stressor controllability, social interaction, and benzodiazepine systems. Pharmacol. Biochem. Behav..

[44] Shirayama Y, Chen ACH, Nakagawa S, Russell DS, Duman RS (2002). Brain-derived neurotrophic factor produces antidepressant effects in behavioral models of depression. J. Neurosci..

[45] Dwivedi Y, Mondal AC, Shukla PK, Rizavi HS, Lyons J (2004). Altered protein kinase a in brain of learned helpless rats: Effects of acute and repeated stress. Biol. Psychiatry.

[46] Oliveira EC, Hunziker MH (2014). Longitudinal investigation on learned helplessness tested under negative and positive reinforcement involving stimulus control. Behav. Processes.

[47] Dwivedi Y, Mondal AC, Payappagoudar GV, Rizavi HS (2005). Differential regulation of serotonin (5HT) 2A receptor mRNA and protein levels after single and repeated stress in rat brain: Role in learned helplessness behavior. Neuropharmacology.

[48] Malberg JE, Duman RS (2003). Cell proliferation in adult hippocampus is decreased by inescapable stress: Reversal by fluoxetine treatment. Neuropsychopharmacology.

[49] Ho YJ, Eichendorff J, Schwarting RK (2002). Individual response profiles of male Wistar rats in animal models for anxiety and depression. Behav. Brain Res..

[50] Desousa NJ, Wunderlich GR, De Cabo C, Vaccarino FJ (1998). Individual differences in sucrose intake predict behavioral reactivity in rodent models of anxiety. Pharmacol. Biochem. Behav..

[51] Koolhaas JM (1999). Coping styles in animals: Current status in behavior and stress-physiology. Neurosci. Biobehav. Rev..

[52] De Boer SF, Buwalda B, Koolhaas JM (2017). Untangling the neurobiology of coping styles in rodents: Towards neural mechanisms underlying individual differences in disease susceptibility. Neurosci. Biobehav. Rev..

[53] Kim JY, Yang SH, Kwon J, Lee HW, Kim H (2017). Mice subjected to uncontrollable electric shocks show depression-like behaviors irrespective of their state of helplessness. Behav. Brain Res..

[54] Meng X, Shen F, Li C, Li Y, Wang X (2016). Depression-like behaviors in tree shrews and comparison of the effects of treatment with fluoxetine and carbetocin. Pharmacol. Biochem. Behav..

[55] Substance Abuse and Mental Health Services Administration (SAMHSA). Key substance use and mental health indicators in the United States: Results from the 2022 National Survey on Drug Use and Health (HHS Publication No. PEP23-07-01-006, NSDUH Series H-58). Center for Behavioral Health Statistics and Quality, Substance Abuse and Mental Health Services Administration (2023). Available on: https://www.samhsa.gov/data/report/2022-nsduh-annual-national-report.

[56] Porsolt RD, Le Pichon M, Jalfre ML (1977). Depression: A new animal model sensitive to antidepressant treatments. Nature.

[57] Pryce CR, Azzinnari D, Spinelli S, Seifritz E, Tegethoff M, Meinlschmidt G (2011). Helplessness: A systematic translational review of theory and evidence for its relevance to understanding and treating depression. Pharmacol. Ther..

[58] Molendijk ML, de Kloet ER (2015). Immobility in the forced swim test is adaptive and does not reflect depression. Psychoneuroendocrinology.

[59] Molendijk ML, de Kloet ER (2019). Coping with the forced swim stressor: Current state-of-the-art. Behav. Brain Res..

[60] De Kloet, E. R. & Molendijk, M. L. Coping with the forced swim stressor: Towards understanding an adaptive mechanism. *Neural Plast.* **2016** (2016). 10.1155/2016/6503162 PMC4806646 27034848

[61] Commons KG, Cholanians AB, Babb JA, Ehlinger DG (2017). The rodent forced swim test measures stress-coping strategy, not depression-like behavior. ACS Chem. Neurosci..

[62] Veldhuis HD, De Korte CC, De Kloet ER (1985). Glucocorticoids facilitate the retention of acquired immobility during forced swimming. Eur. J. Pharmacol..

[63] De Kloet ER, De Kock S, Schild V, Veldhuis HD (1988). Antiglucocorticoid RU 38486 attenuates retention of a behaviour and disinhibits the hypothalamic-pituitary adrenal axis at different brain sites. Neuroendocrinology.

[64] West AP (1990). Neurobehavioral studies of forced swimming: The role of learning and memory in the forced swim test. Prog. Neuropsychopharmacol. Biol. Psychiatry.

[65] Willner P, Towell A, Sampson D, Sophokleous S, Muscat RA (1987). Reduction of sucrose preference by chronic unpredictable mild stress, and its restoration by a tricyclic antidepressant. Psychopharmacology.

[66] Der-Avakian A, Markou A (2012). The neurobiology of anhedonia and other reward-related deficits. Trends Neurosci..

[67] Christianson JP (2010). 5-hydroxytryptamine 2C receptors in the basolateral amygdala are involved in the expression of anxiety after uncontrollable traumatic stress. Biol. Psychiatry.

[68] Fried EI, Nesse RM (2015). Depression is not a consistent syndrome: An investigation of unique symptom patterns in the STAR*D study. J. Affect. Disord..

[69] Maglanoc LA (2019). Data-driven clustering reveals a link between symptoms and functional brain connectivity in depression. Biol. Psychiatry Cogn. Neurosci. Neuroimaging.

[70] Einat H, Ezer I, Kara NZ, Belzung C (2018). Individual responses of rodents in modelling of affective disorders and in their treatment: Prospective review. Acta Neuropsychiatr..

[71] Wood SK, Walker HE, Valentino RJ, Bhatnagar S (2010). Individual differences in reactivity to social stress predict susceptibility and resilience to a depressive phenotype: Role of corticotropin-releasing factor. Endocrinology.

[72] Armario A, Nadal R (2013). Individual differences and the characterization of animal models of psychopathology: A strong challenge and a good opportunity. Front. Pharmacol..

[73] Ebner K, Singewald N (2017). Individual differences in stress susceptibility and stress inhibitory mechanisms. Curr. Opin. Behav. Sci..

[74] Dopfel D (2019). Individual variability in behavior and functional networks predicts vulnerability using an animal model of PTSD. Nat. Commun..

[75] Careaga MBL, Girardi CEN, Suchecki D (2019). Variability in response to severe stress: Highly reactive rats exhibit changes in fear and anxiety-like behavior related to distinct neuronal co-activation patterns. Behav. Brain Res..

[76] MacKillop J (2016). The latent structure of impulsivity: Impulsive choice, impulsive action, and impulsive personality traits. Psychopharmacology.

[77] Ruggiero RN, Marques DB, Rossignoli MT, De Ross JB, Prizon T, Beraldo IJS, Leite JP (2024). Dysfunctional hippocampal-prefrontal network underlies a multidimensional neuropsychiatric phenotype following early-life seizure. eLife.

[78] McIlwain KL, Merriweather MY, Yuva-Paylor LA, Paylor R (2001). The use of behavioral test batteries: Effects of training history. Physiol. Behav..

[79] Lad HV, Liu L, Paya-Cano JL, Parsons MJ, Kember R, Fernandes C, Schalkwyk LC (2010). Behavioural battery testing: Evaluation and behavioural outcomes in 8 inbred mouse strains. Physiol. Behav..

[80] Blokland A, Ten Oever S, Van Gorp D, Van Draanen M, Schmidt T, Nguyen E, Klinkenberg I (2012). The use of a test battery assessing affective behavior in rats: Order effects. Behav. Brain Res..

[81] Neuwirth LS, Verrengia MT, Harikinish-Murrary ZI, Orens JE, Lopez OE (2022). Under or absent reporting of light stimuli in testing of anxiety-like behaviors in rodents: The need for standardization. Front. Mol. Neurosci..

[82] Hiew LF, Khairuddin S, Aquili L, Koh J, Fung ML, Lim WL, Lim LW (2020). Behavioural responses of anxiety in aversive and non-aversive conditions between young and aged Sprague-Dawley rats. Behav. Brain Res..

[83] Kerr DM, Gilmartin A, Roche M (2016). Pharmacological inhibition of fatty acid amide hydrolase attenuates social behavioural deficits in male rats prenatally exposed to valproic acid. Pharmacol. Res..

[84] Garcia AMB, Cardenas FP, Morato S (2005). Effect of different illumination levels on rat behavior in the elevated plus-maze. Physiol. Behav..

[85] Robinson L, Spruijt B, Riedel G (2018). Between and within laboratory reliability of mouse behaviour recorded in home-cage and open-field. J. Neurosci. Methods.

[86] Hetzler BE, McLester-Davis LW, Tenpas SE (2019). Methylphenidate and alcohol effects on flash-evoked potentials, body temperature, and behavior in Long-Evans rats. Alcohol.

[87] Zanos P, Moaddel R, Morris PJ, Georgiou P, Fischell J, Elmer GI, Gould TD (2016). NMDAR inhibition-independent antidepressant actions of ketamine metabolites. Nature.

[88] Fitzgerald PJ, Yen JY, Watson BO (2019). Stress-sensitive antidepressant-like effects of ketamine in the mouse forced swim test. PLoS One..

[89] Pijlman FT, Herremans AH, van de Kieft J, Kruse CG, van Ree JM (2003). Behavioural changes after different stress paradigms: Prepulse inhibition increased after physical, but not emotional stress. Eur. Neuropsychopharmacol..

[90] Budaev SV (2010). Using principal components and factor analysis in animal behaviour research: Caveats and guidelines. Ethology.

[91] Gorsuch RL (1983). Factor Analysis.

[92] Blanchard DC, Shepherd JK, Carobrez ADP, Blanchard RJ (1991). Sex effects in defensive behavior: Baseline differences and drug interactions. Neurosci. Biobehav. Rev..

[93] Drysdale AT, Patel GH (2022). A dynamic approach to depression treatment prediction. Biol. Psychiatry.

[94] Zhang W, Sweeney JA, Bishop JR, Gong Q, Lui S (2023). Biological subtyping of psychiatric syndromes as a pathway for advances in drug discovery and personalized medicine. Nat. Ment. Health.

[95] Cardenas F, Lamprea MR, Morato S (2001). Vibrissal sense is not the main sensory modality in rat exploratory behavior in the elevated plus-maze. Behav. Brain Res..

[96] Tejada, J., Chaim, K. T., & Morato, S. X-PloRat: A software for scoring animal behavior in enclosed spaces. *Psic Teor e Pesq*. **33** (2018).

[97] Seibenhener ML, Wooten MC (2015). Use of the open field maze to measure locomotor and anxiety-like behavior in mice. J. Vis. Exp..

[98] Sturman O, Germain PL, Bohacek J (2018). Exploratory rearing: A context-and stress-sensitive behavior recorded in the open-field test. Stress.

[99] Van Dijken HH, Mos J, van der Heyden JA, Tilders FJ (1992). Characterization of stress-induced long-term behavioural changes in rats: Evidence in favor of anxiety. Physiol. Behav..

[100] Prut L, Belzung C (2003). The open field as a paradigm to measure the effects of drugs on anxiety-like behaviors: A review. Eur. J. Pharmacol..

[101] Slattery DA, Cryan JF (2012). Using the rat forced swim test to assess antidepressant-like activity in rodents. Nat. Protoc..

[102] Berton O (2006). Essential role of BDNF in the mesolimbic dopamine pathway in social defeat stress. Science.

[103] Lukas M (2011). The neuropeptide oxytocin facilitates pro-social behavior and prevents social avoidance in rats and mice. Neuropsychopharmacology.

[104] Krishnan V, Han MH, Graham DL, Berton O, Renthal W, Russo SJ, Nestler EJ (2007). Molecular adaptations underlying susceptibility and resistance to social defeat in brain reward regions. Cell..

[105] Golden SA, Covington HE, Berton O, Russo SJ (2011). A standardized protocol for repeated social defeat stress in mice. Nat. Protoc..

[106] Walf AA, Frye CA (2007). The use of the elevated plus maze as an assay of anxiety-related behavior in rodents. Nat. Protoc..

[107] Belda X, Márquez C, Armario A (2004). Long-term effects of a single exposure to stress in adult rats on behavior and hypothalamic–pituitary–adrenal responsiveness: Comparison of two outbred rat strains. Behav. Brain Res..

[108] Bevins RA, Besheer J (2006). Object recognition in rats and mice: A one-trial non-matching-to-sample learning task to study ‘recognition memory’. Nat. Protoc..

[109] Fendt M, Koch M (2013). Translational value of startle modulations. Cell Tissue Res..

[110] Liu MY (2018). Sucrose preference test for measurement of stress-induced anhedonia in mice. Nat. Protoc..

[111] Vollmayr B, Henn FA (2001). Learned helplessness in the rat: Improvements in validity and reliability. Brain Res. Protoc..

[112] Amat J, Baratta MV, Paul E, Bland ST, Watkins LR, Maier SF (2005). Medial prefrontal cortex determines how stressor controllability affects behavior and dorsal raphe nucleus. Nat. Neurosci..

[113] The MathWorks Inc. MATLAB version: 9.13.0 (R2022b), Natick, Massachusetts: The MathWorks Inc. https://www.mathworks.com (2022).

[114] Koo TK, Li MY (2016). A guideline of selecting and reporting intraclass correlation coefficients for reliability research. J. Chiropr. Med..

