# Supplementary material for: Multidimensional behavioral profiles associated with resilience and susceptibility after inescapable stress
Source: Sci Rep. 2024 Apr 27;14:9699. doi: 10.1038/s41598-024-59984-7 (PMC11055923; doi:10.1038/s41598-024-59984-7)
Supplement: Supplementary file 1 — Supplementary Information. [file 41598_2024_59984_MOESM1_ESM.docx]

**Multidimensional behavioral profiles associated with resilience and susceptibility after inescapable stress**

**Running title: Multidimensional stress coping behavioral profiles**

**Benedito Alves de Oliveira-Júnior^1*^, Danilo Benette Marques^1^, Matheus Teixeira Rossignoli^1^, Tamiris Prizon^1^, João Pereira Leite^1^, Rafael Naime Ruggiero^1*^**

^1^Department of Neuroscience and Behavioral Sciences, Ribeirão Preto Medical School, University of São Paulo, Ribeirão Preto, São Paulo, Brazil.

**Supplementary Material**

Supplementary Figures and Legends

**Figure S1. Stronger intra-test than inter-test behavioral correlations.** (**A**) Feature clustering by sign-independent pattern similarity (minimum Euclidean distance) clearly separates the behavioral tests (each test is indicated by a color in the bar below). Dashed lines in the correlation matrix indicate clustered variables of the same test. (**B-C**) Factor analysis for seven factors effectively captures latent covariation corresponding to the six behavioral tests. Each factor shows high loadings only across variables of the same test. Variables are ordered by the experimental design.
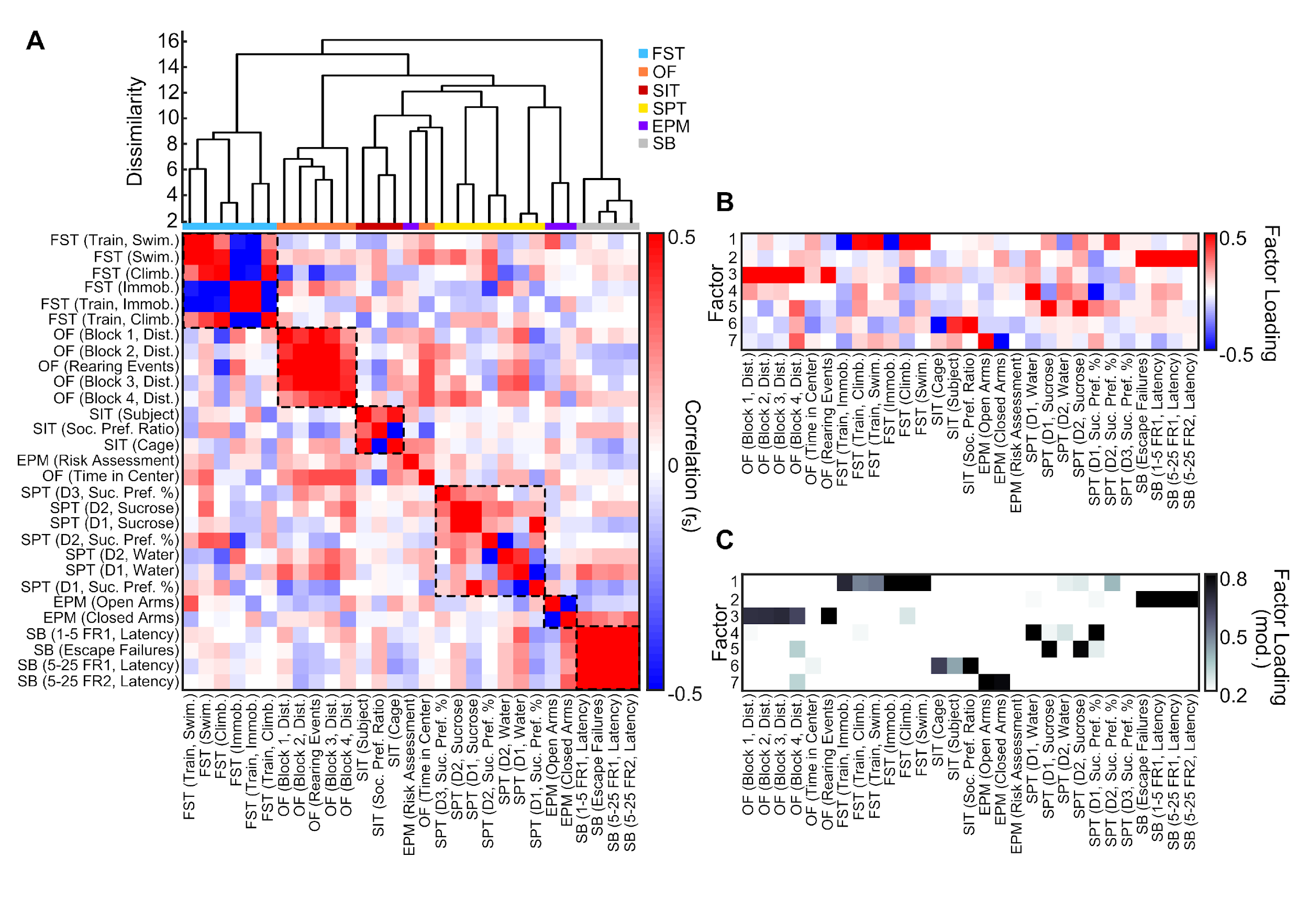


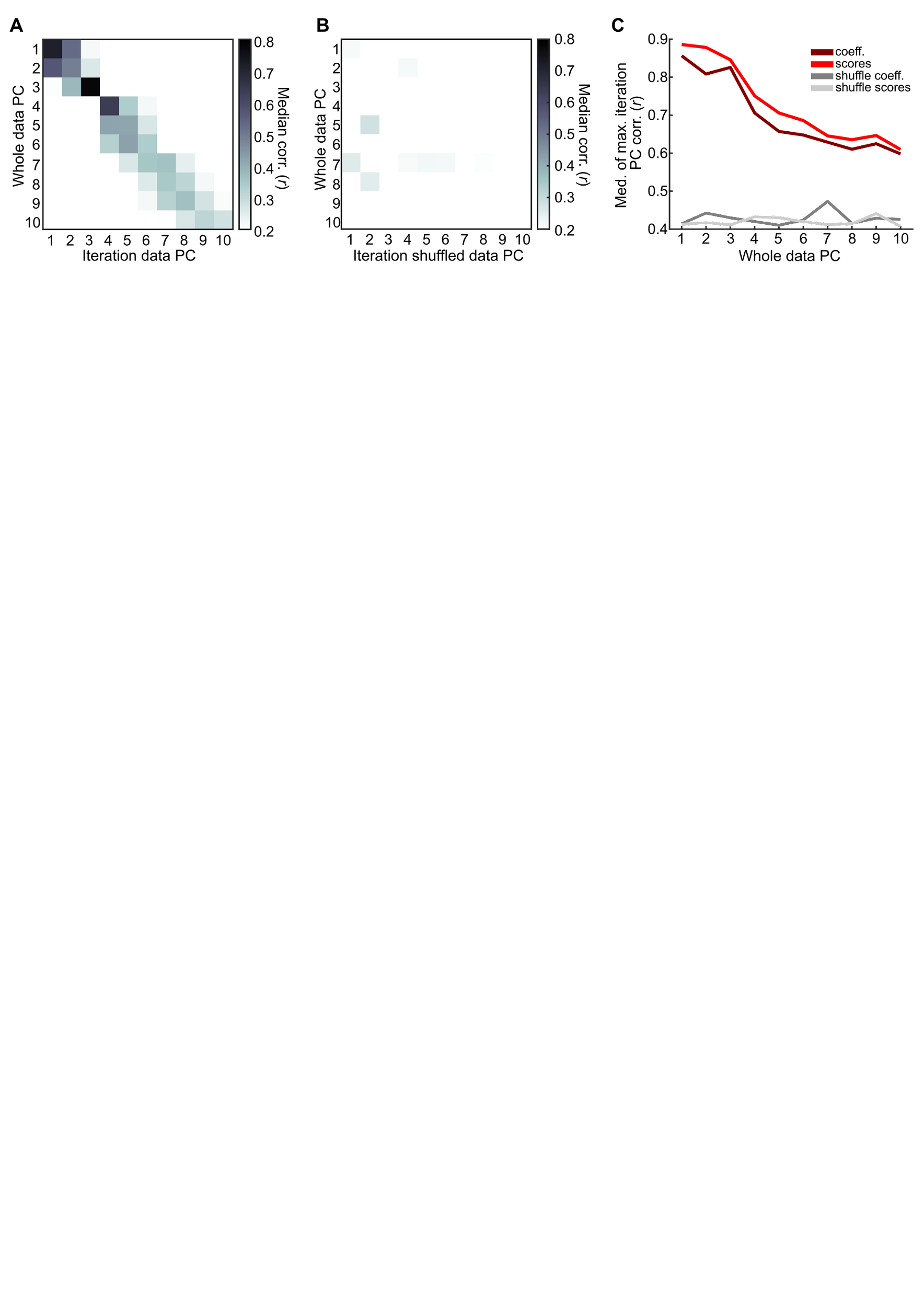


**Figure S2. Robustness of multidimensional patterns of covariation.** (**A**) The PCs from the whole data also emerge in partitioned data with fewer observations. The heatmap indicates that the PC’s coefficients are frequently correlated to PCs of partitioned data (randomly selected 70% of observations across 10^4^ iterations). Note that each PC, but especially the first ones, shows great correlation with PCs of close ranking of variance explained. (**B**) There are no clear correlations between whole data PCs to that from shuffled data. (**C**) By choosing the most similar PC to the whole data PC for every iteration, we observed a frequent high correlation of the initial PC’s, for both scores and coefficients, that does not emerge in the shuffled data.

**Figure S3. Main-variables PCA revealing that increased time in the open arms of the EPM, reduced sucrose preference, and increased escape failures in the SB, consistently distinguish susceptible from resilient rats.** **(A)** Collinearity evaluation. Variance inflation factor (VIF) values indicate low interdependence between the selected variables. (**B**) Explained variance of the Principal Components. (**C**) Scores of individuals by group on each principal component. The groups are distinguished by PC2 (IS vs. NS, H vs. NH) and PC3 (H vs. NH). Student t-test or Wilcoxon rank-sum test, ***p < 0.001, ****p < 0.0001. (**D**) Coefficients of principal components related to behavioral variables. **(E**) The scores of individuals were projected onto the axes space of the first three principal components. Individuals categorized between IS and NS groups (left) or H and NH clusters (right).
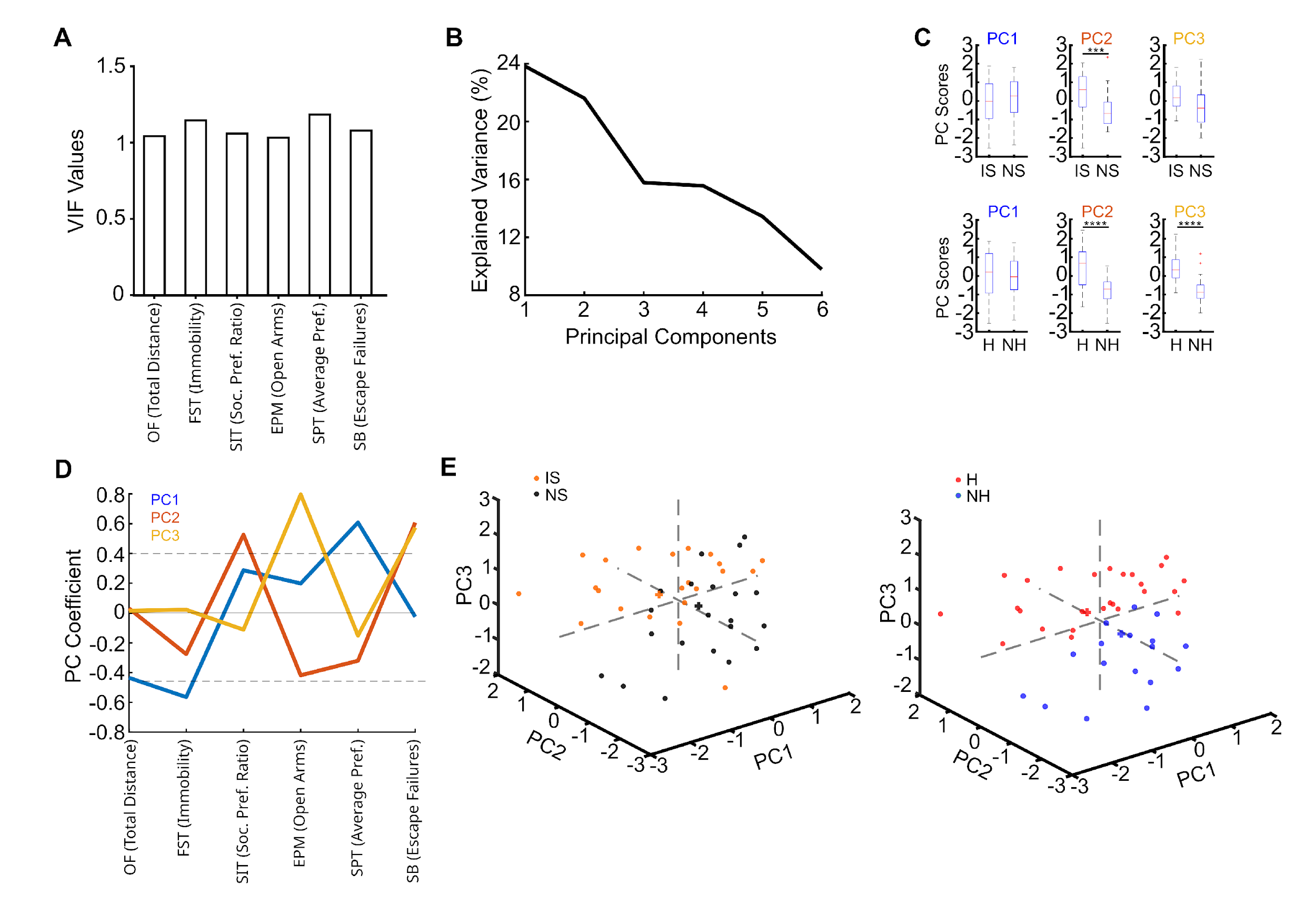


**Figure S4. Distinct clustering algorithms yield similar behavioral profiles.** (**A**) Silhouette values indicate six and seven clusters as appropriate for k-means clustering. (**B**) Specifically, four to six clusters can discriminate between NS vs. IS (Chi-squared test p-value). (**C**) Silhouette values for six clusters. (**D**) Clusters show a spectrum of distinction between proportions of NS vs. IS individuals. (**E**) Multidimensional behavioral profiles identified by k-means (six clusters, left) and hierarchical (seven clusters, right) clustering. Note the correspondence (indicated by similar color) of the multidimensional profiles between the two distinct clustering algorithms. Also note the emergence of an identical grouping as the HC Cluster 7 characterized by a generalized susceptibility profile. The HC clusters are the same as in Figure 5. Variables are ordered by resilience and susceptibility, then by experimental design. Lines and shaded boundaries represent the mean ± SEM.
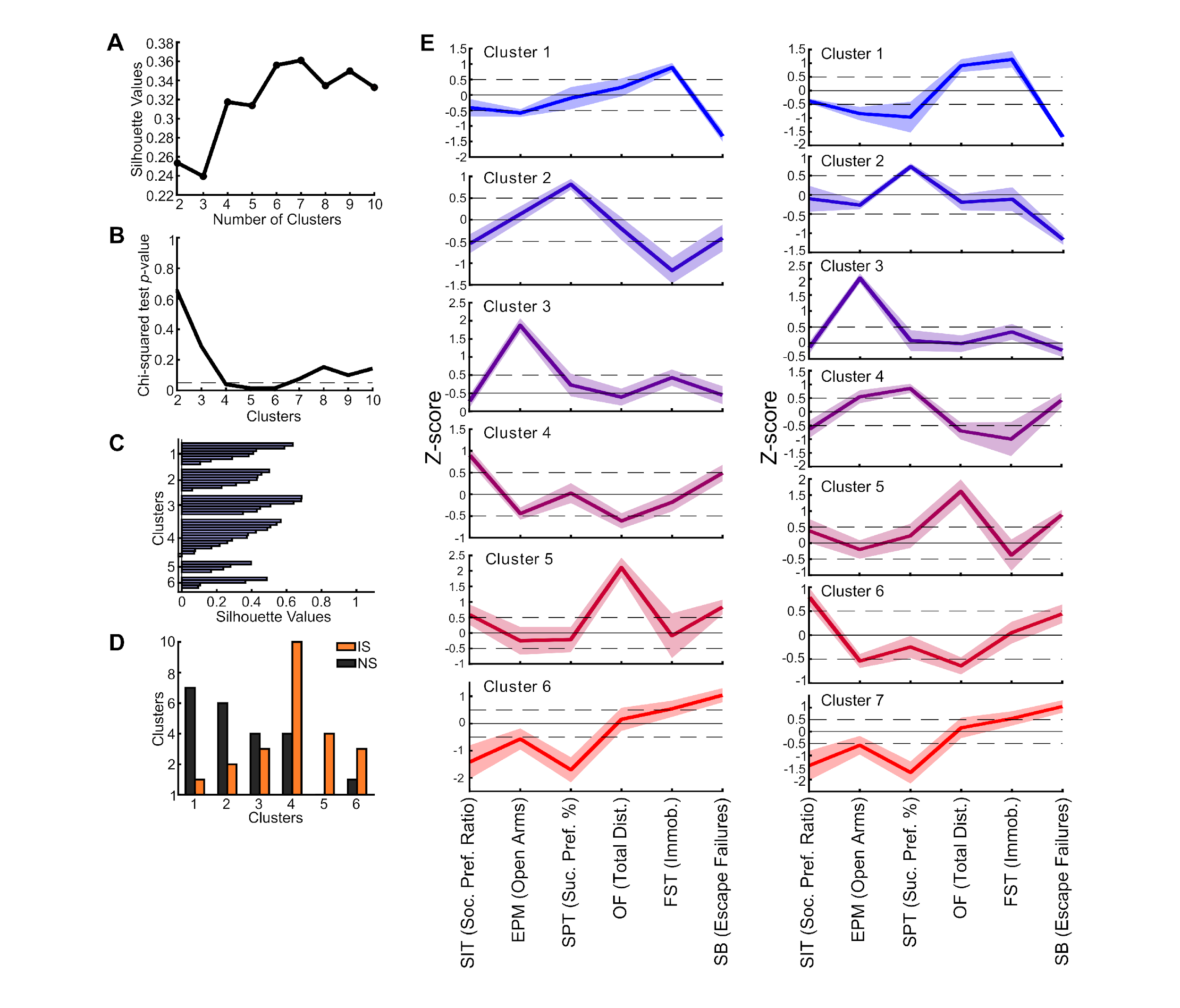


**Figure S5.** Literature reported behavioral effects of acute inescapable shocks. All comparisons are against the respective control groups. Green boxes indicate when the respective behavioral tests in our study were conducted.
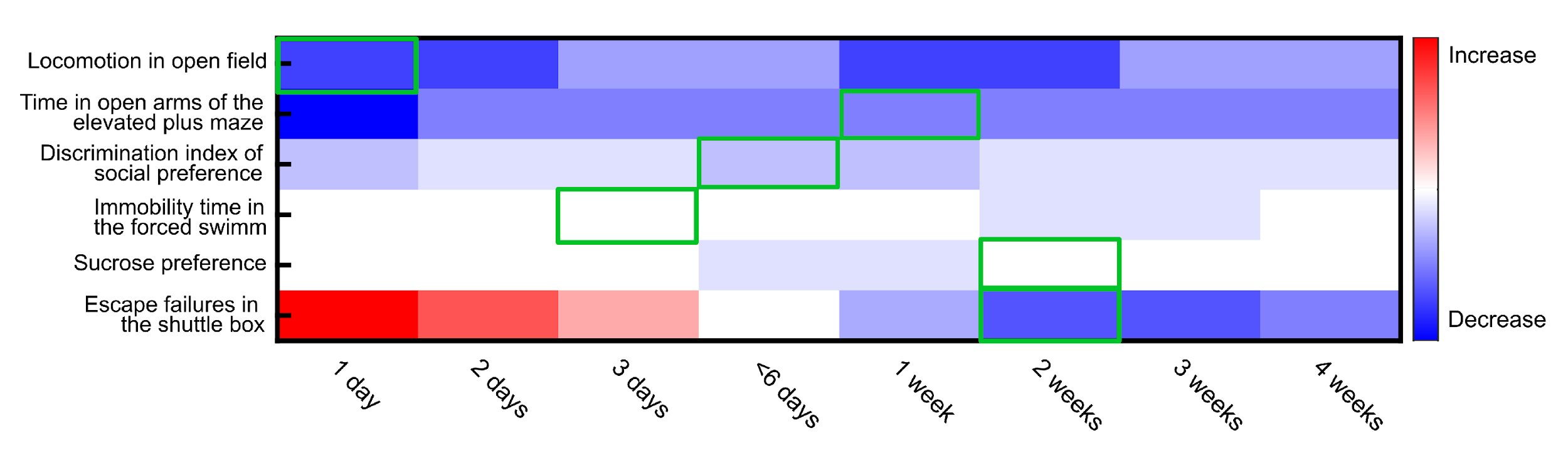


| **Test** | **Resilience-related variables** | **Susceptibility-related variables** | **References** |
| --- | --- | --- | --- |
| OF | Time in center | Distance traveled (total and per block); Rearing events | Seibenhener & Wooten, 2015; Prut & Belzung, 2003; Sturman et al., 2018 |
| FST | Climbing; Swimming | Immobility | Porsolt et al., 1977; Slattery & Cryan, 2012 |
| SIT | Time on subject; social preference ratio | Time on cage | Krishnan et al., 2007; Lukas et al., 2011 |
| EPM | Time in open arms | Time in closed arms. Risk assessment | Walf & Frye, 2007 |
| SPT | Sucrose consumption; Sucrose preference | Water consumption | Liu et al., 2018 |
| SB |  | Escape latency (1-5 FR1 and 5-25 FR1, 5-25 FR2); Escape failures | Vollmayr et al., 2001; Amat et al., 2005; Marques et al., 2022 |

**Supplementary Table 1**. Literature-based classification of resilience- and susceptibility-related behavioral variables across tests.

| **Behavioral test** | **1 Day** | **2 Days** | **3 Days** | **< 6 Days** | **1 Week** | **2 Weeks** | **3 Weeks** | **4 Weeks** | **Authors** |
| --- | --- | --- | --- | --- | --- | --- | --- | --- | --- |
| OF | ↓ | ↓ |  | ↓ | ↓ | ↓ |  |  | Van Dijken et al. (1992a) |
| OF |  |  |  |  |  |  |  | ↓ | Van Dijken et al. (1992b) |
| OF |  |  |  |  | ↓ | ↓ |  |  | Kinn Rød et al. (2012) |
| FST |  |  |  |  |  | ↓ |  |  | Van Dijken et al. (1992b) |
| SIT | ↓ |  |  |  |  |  |  |  | Christianson et al. (2010) |
| EPM |  |  |  |  | ↓ | ↓ | ↓ |  | Kinn Rød et al. (2012) |
| EPM |  |  |  |  |  | ↑ |  |  | Belda et al. (2004) |
| EPM | ↓ |  |  |  |  |  |  |  | Steenbergen et al. (1990) |
| EPM | ↓ |  | ↓ |  |  |  |  |  | Steenbergen et al. (1991) |
| SPT |  |  |  | ↓ |  |  |  |  | Kinn Rød et al. (2012) |
| SB | ↑ |  |  |  |  |  |  |  | Shirayama et al. (2002) |
| SB |  |  |  |  |  | ↓ | ↓ |  | Van Dijken et al. (1992b) |
| SB |  | ↑ |  |  |  |  |  |  | Dwivedi et al. (2004) |
| SB |  | ↑ |  |  |  |  |  |  | Dwivedi et al. (2005) |

**Supplementary Table 2.** Table containing the data and references for Figure S5.
